# Simultaneous Quantification of Free Cholesterol, Cholesteryl Esters, and Triglycerides without Ester Hydrolysis by UHPLC Separation and In-Source Collision Induced Dissociation Coupled MS/MS

**DOI:** 10.1007/s13361-017-1756-2

**Published:** 2017-08-11

**Authors:** Michael S. Gardner, Lisa G. McWilliams, Jeffrey I. Jones, Zsuzsanna Kuklenyik, James L. Pirkle, John R. Barr

**Affiliations:** Division of Laboratory Sciences, National Center for Environmental Health, Centers for Disease Control and Prevention, Chamblee Campus, Atlanta, GA 30341 USA

**Keywords:** Collision induced dissociation, Triglyceride, Cholesterol, Lipidomics, Lipoproteins

## Abstract

We demonstrate the application of in-source nitrogen collision-induced dissociation (CID) that eliminates the need for ester hydrolysis before simultaneous analysis of esterified cholesterol (EC) and triglycerides (TG) along with free cholesterol (FC) from human serum, using normal phase liquid chromatography (LC) coupled to atmospheric pressure chemical ionization (APCI) tandem mass spectrometry (MS/MS). The analysis requires only 50 μL of 1:100 dilute serum with a high-throughput, precipitation/evaporation/extraction protocol in one pot. Known representative mixtures of EC and TG species were used as calibrators with stable isotope labeled analogs as internal standards. The APCI MS source was operated with nitrogen source gas. Reproducible in-source CID was achieved with the use of optimal cone voltage (declustering potential), generating FC, EC, and TG lipid class-specific precursor fragment ions for multiple reaction monitoring (MRM). Using a representative mixture of purified FC, CE, and TG species as calibrators, the method accuracy was assessed with analysis of five inter-laboratory standardization materials, showing –10% bias for Total-C and –3% for Total-TG. Repeated duplicate analysis of a quality control pool showed intra-day and inter-day variation of 5% and 5.8% for FC, 5.2% and 8.5% for Total-C, and 4.1% and 7.7% for Total-TG. The applicability of the method was demonstrated on 32 serum samples and corresponding lipoprotein sub-fractions collected from normolipidemic, hypercholesterolemic, hypertriglyceridemic, and hyperlipidemic donors. The results show that in-source CID coupled with isotope dilution UHPLC-MS/MS is a viable high precision approach for translational research studies where samples are substantially diluted or the amounts of archived samples are limited.

**Electronic supplementary material:**

The online version of this article (doi:10.1007/s13361-017-1756-2) contains supplementary material, which is available to authorized users.

**Graphical Abstract:**
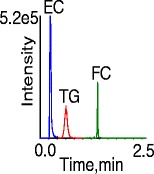
ᅟ

## Introduction

Lipoproteins are dynamic lipid-protein assemblies that function as transporters of various biomolecules circulating in blood [1]. The traditional metrics of lipoprotein levels in serum or plasma is total cholesterol (Total-C) and total triglyceride (Total-TG) concentrations. With the emergence of new biomarkers and development of new drug therapies for lipid metabolism-related disorders, there is increasing reliance on “big data” surveys, where several thousands of archive samples are analyzed [2]. These specimens are usually collected in expensive temporal studies and are only available in very limited amounts, and require development of sensitive, multiplexed, high-throughput, automated workflows that also meet quality control requirements of clinical measurements. Meeting these challenges means application of advanced techniques to traditional biomarkers as well.

The typical clinical analyses of Total-TG and Total-C are performed by colorimetric assays based on the detection of hydrogen peroxide. The hydrogen peroxide is produced through a series of four enzymatic reactions in case of Total-TG, and two enzymatic reactions in case of Total-C measurements. The determination of free and esterified cholesterol (FC and EC), along with the measurement of TG (without free glycerol) requires at least two serum aliquots, 60–120 μL in total when performed manually or 200–1000 μL when performed on a clinical analyzer. One aliquot (or two parallel aliquots) is needed for FC and EC analysis, based on two absorbance readings. A second sample aliquot (or two additional in parallel) is needed for the free glycerol and TG measurements, based on another two absorbance readings. As an alternative, gas chromatography (GC) with mass spectrometry (MS) detection has been used. In addition to liquid–liquid extraction, GC-MS methods require labor-intensive extraction and derivatization considered impractical for routine analysis, and are only used for characterization of reference standard materials [3–6].

Recently, the potential of coupling hydrophilic interaction (HILIC) and normal phase (NP) liquid chromatography (LC) with tandem MS (MS/MS) detection was demonstrated for quantitative analysis of lipids [7, 8]. Using HILIC or NP LC, each lipid class is generally eluted under the same chromatographic peak with the same retention time, with little regard for differences among the individual species in the length of the acyl carbon chains, or number/position of double bounds. The MS instruments used in a variety of MS/MS scanning modes allow both lipid class-selective and species-selective detection and quantification. For the analysis of nonpolar lipids, an important advantage of the lipidomic approach is that EC and TG classes have similar elution behavior for analysis in the same LC-MS/MS injection run. At the same time, elution behaviors of FC, EC, and TG are different enough to be separated and detected separately. Using atmospheric pressure chemical ionization (APCI), both FC and EC species yield [cholesterol + H – H_2_O]^+^ ions in the LC-MS interface for sensitive multiple reaction monitoring (MRM). In previously reported methods, individual TG species are detected by hundreds of MRM chromatograms. Even when all the TG MRM chromatograms are added up to one LC-MS/MS peak, the S/N is still quite low, and sufficient amount of sample (i.e., 50–200 μL of serum) has to be extracted and preconcentrated by liquid–liquid extraction before LC-MS/MS analysis. In addition, the liquid–liquid extraction requires manual separation of aqueous and organic phases.

In this work, we demonstrate an LC-MS/MS based approach for simultaneous quantification of FC along with EC and TG in serum. The unique features of this method are the high-throughput, 2.5 min/injection LC-MS/MS run, and the enhanced TG sensitivity, achieved by generation of a single TG class-specific CID fragment as a parent ion for MS/MS detection (instead of hundreds of individual MRMs). Additionally, we have identified a series of low mass fragment ions (<150 *m*/*z*) that can be generated from TGs at elevated collision energies, either in the Q2 collision cell or in-source. Some of these fragments appear to be common to TGs as a class regardless of fatty acid species.

Because of the enhanced TG sensitivity, a relatively small amount of serum (<1 μL) was enough for TG, EC, and FC quantification. The small sample volume allowed the extraction of non-polar lipids to be performed in a high-throughput 96-well format. Each sample was extracted in a single well, “in one-pot”, without the need for manual liquid phase separation or sample transfer before LC-MS/MS analysis. The method was applied to whole serum concentration measurements of Total-FC, Total-EC, and Total-TG in 30 serum samples collected from hyperlipidemic donors. The LC-MS/MS results were compared with concentrations obtained by a colorimetric clinical analyzer. The sensitivity of the method allowed the high-throughput analysis of lipoprotein sub-fractions, where 50 μL of serum samples from 30 individuals were separated into 40 size fractions per serum sample, using asymmetric flow field-flow fractionation (AF4).

## Experimental

All organic solvents were HPLC grade (except nonane, which was 99% pure reagent grade) and purchased from Thermo Fisher Scientific (Waltham, MA, USA). Labeled d_7_-cholesterol was purchased from Sigma-Aldrich (USA). Cholesteryl-d_7_-palmitate was purchased from Avanti Polar Lipids, (Alabaster, AL, USA). Labeled d_98_-tripalmitin was purchased from CDN Isotopes, (Pointe-Claire, Quebec, Canada). Two serum reference materials, SRM 1951c Level 1 and Level 2, were provided by the National Institute of Standards and Technology (NIST, Gaithersburg, MD, USA). Five value assigned reference materials (701, 707, 713, 801, and 813) were provided by the Lipid Standardization Program (LSP) at the Centers for Disease Control (CDC). Four units of frozen human serum from de-identified individuals were purchased from Interstate Blood Bank (Memphis, TN, USA) and were used for the preparation of a quality control (QC) pool. After mixing, the pool was distributed into 1 mL aliquots and stored at –80 °C. The 32 de-identified serum samples from normolipidemic (N = 11), hypercholesterolemic (N = 6), hypertriglyceridemic (N = 9), and hyperlipidemic donors (N = 6) were purchased frozen from Bioreclamation IVT (New York City, NY, USA), stored at –80 °C until analysis. All individual donor samples were viral tested before shipment.

### Size Fractionation of Serum Lipoproteins

Asymmetric flow field-flow fractionation (AF4) was optimized to separate 50 μL serum into 40 size fractions that were each 250 μL. The carrier fluid was 10 mM sodium bicarbonate and 150 mM sodium chloride in deionized water, pH 7.4. The average hydrodynamic size in each fraction was determined based on AF4 retention time and dynamic light scattering (DLS) measurements using a Dynapro plate reader (Wyatt Technologies, Santa Barbara, CA, USA). Details of the AF4 separation are described in a previous publication and in Supporting Information [9].

### Preparation of Calibrators and Internal Standard Spiking Solutions

Because we observed that all pure EC standards ionized with the same ionization efficiency (see below), we used only cholesteryl palmitate as the external calibrator for the EC analyte group. For TG standards we found significant differences in ionization efficiency. Therefore, a mixture of triolein, tripalmitin, and trilinolein in a ratio of 514:313:173, reflective of the typical ratio in humans, was used as external calibrator for the TG analyte group. The internal standard (IS) spiking mix was prepared in ethanol; containing 0.033 mg/dL d_7_-cholesterol (IS for FC), 0.098 mg/dL cholesteryl-d_7_-palmitate (IS for EC), and 0.125 mg/dL d_98_-tripalmitin (IS for TG).

### Sample Preparation

A 50 μL aliquot of each AF4 fraction, or 1:100 diluted serum in AF4 buffer, was placed on a 96-wellplate, along with 50 μL aliquots of the calibrator dilution series. To all wells, 200 μL of the IS/ethanol solution was added by a Biomek FX liquid handler (Beckman Coulter, Indianapolis, IN, USA). The plate was vortex-mixed on an orbital shaker at 500 rpm for 2 min. Samples were evaporated under a stream of air, with 60 °C heating plate temperature for approximately 30 min until dryness was observed by visual inspection. Given the use of normal-phase LC, complete evaporation was important. The elevated temperature was necessary to accomplish this as rapidly as possible. The 60 °C applies to the heating plate only. During evaporation, the actual temperature in the sample was reduced because of evaporative cooling. The temperature of a sample well was continuously monitored during a typical evaporation step by placing a Type K thermocouple at the bottom of the well. The temperature remained between 30 and 40 °C during the entire process. Removing the plates from heat immediately upon drying was important to minimize lipid oxidation. Samples were reconstituted in 50 μL of nonane and the plate was vortex-mixed on an orbital shaker at 500 rpm for 2 min. The plate was covered with a heat-sealing foil mat and centrifuged for 3 min at 3700 rpm prior to analysis by UHPLC-MS/MS.

As a comment, when we used less diluted serum, we observed that the pellet sometimes came loose from the bottom of the wells after dry down. We do not have evidence that this impacted reproducibility, but it was a concern that powdering from the pellet would cause cross-contamination. For this reason, we do not recommend the use of this extraction method in the well plate format for less diluted or undiluted serum.

### Analytical UHPLC Separation

The UHPLC system was an Agilent 1290 (Agilent Technologies, Santa Clara, CA, USA). From each sample, 6 μL was injected. The column was a Kinetex HILIC 1.7 μm, 2.1 × 50 mm (Phenomenex, Torrance, CA, USA). Mobile phase A was hexanes with 0.05% isopropanol. Mobile phase B was hexanes with 5% ethanol and 0.05% isopropanol. Both contained 0.05% IPA for more stable UHPLC pump performance. The mobile phase flow rate was 600 μL/min. The gradient program started at 2% B, increased to 4% B over 0.5 min, then to 80% B over 1.0 min, returned to 2% B over 0.1 min, and finally held at 2% B for 0.9 min to re-equilibrate the column for the subsequent analysis. The total run time was 2.5 min.

### Mass Spectrometry

The mass spectrometer was a hybrid triple quadrupole/linear ion trap Sciex 4000 QTrap (Sciex, Framingham, MA, USA). The Sciex MS heated nebulizer (APCI) interface, normally operated with air as the nebulizer gas, was used with nitrogen because of the flammable nature of the volatile organic LC eluent. The APCI source was operated with a nebulizer gas pressure of 70 psi and a curtain gas pressure of 10 psi, in positive ionization mode, with 4 μA nebulizer current, 325 °C source temperature, and medium nitrogen collision gas pressure. The MRM parameters, declustering potentials (DP, also called cone voltage), collision energy potentials (CE), and collision cell exit potentials (CXP) are summarized in Table 1.

**Table 1:** MS Acquisition Time Segments with Precursor and Product Ion Masses, Compound-Specific Declustering Potentials (DP), Collision Energy Potentials (CE), Collision Cell Exit Potentials (CXP) and Typical Fragment Ion Ratios from Various Matrices

Period start time (min)	Compound	Precursor ion *m*/*z*	Fragment Ion						
			Product *m*/*z*	Dwell time (ms)	DP (V)	CE (V)	CXP (V)	Quantitation (Q) Confirmation (C)	C/Q ion ratio
0.0	Cholesteryl ester	369	161	25	0	15	5.0	Q	0.39 (±0.05)
			81	25	0	20	5.0	C	
	Cholesteryl-d_7_ palmitate (IS)	376	161	25	0	15	5.0	Q	0.31 (±0.01)
			81	25	0	20	5.0	C	
0.4	Triglycerides	95	67	40	262	20	10.7	Q	0.51 (±0.04)
			55	40	262	26	8.8	C	
	d_98_-Tripalmitin (IS)	98	50	40	262	15	15.0	Q	0.65 (±0.05)
		106	74	40	262	25	15.0	C	
1.0	Free cholesterol	369	161	25	10	15	5.0	Q	0.67 (±0.06)
			81	25	10	20	5.0	C	
	d_7_-Cholesterol (IS)	376	161	25	10	15	5.0	Q	0.64 (±0.03)
			81	25	10	20	5.0	C	

As an interesting technical note, the Sciex 4000 QTrap needed extra pause time to switch between low (0 or 10) and high (262) V DP settings. For this reason, when acquiring all MRMs in a single time segment, we had to include extra transitions into the actual MRM acquisition table between FC/EC and TG MRMs. Without adding the extra MRM transitions, the first MRM between low and high DP values exhibited excessive noise. These extra transitions were not necessary if the low and high DP transitions were in separate time segments (Table 1). However, time segmented acquisition required baseline resolution of the EC and TG peaks, which was achieved by gradient UHPLC separation.

## Results and Discussion

### Method Development

#### Sample Preparation

The simultaneous analysis of FC, EC, and TG species required careful method design from extraction to LC-MS/MS detection. The external calibrator mix and IS spiking mix were designed to simulate the nonpolar lipid composition of typical serum samples. The entire sample preparation was achieved in one 96-wellplate. Addition of ethanol spiked with the IS mix allowed precipitation of plasma proteins and removal of water by evaporation. Several solvents were tested for extraction of the analytes from the dry pellets. Nonane was selected because it served as an optimal LC injection matrix and it gave ~70% analyte recovery, which was corrected by the use of isotopically labeled internal standards.

Although a stream of dry air was used in this work, as it was more readily available in our lab for simultaneously drying multiple well plates, nitrogen is more commonly utilized in lipid analysis to minimize oxidation of the lipids. We evaluated the observed differences in measured concentrations of our analytes using nitrogen for evaporation versus air on six randomly selected whole plasma samples analyzed in replicate (n = 5). The observed differences are given in Supplementary Figure S1. A small but consistent positive bias is observed for EC, averaging +4.1% across samples, when using air as opposed to nitrogen. Biases are smaller and not observed in a consistent direction for TG and FC. This method can be used with nitrogen without sacrificing throughput, if there is a ready supply of high purity nitrogen available at approximately 25 L/min per well plate.

#### LC Separation

**Figure 1:**
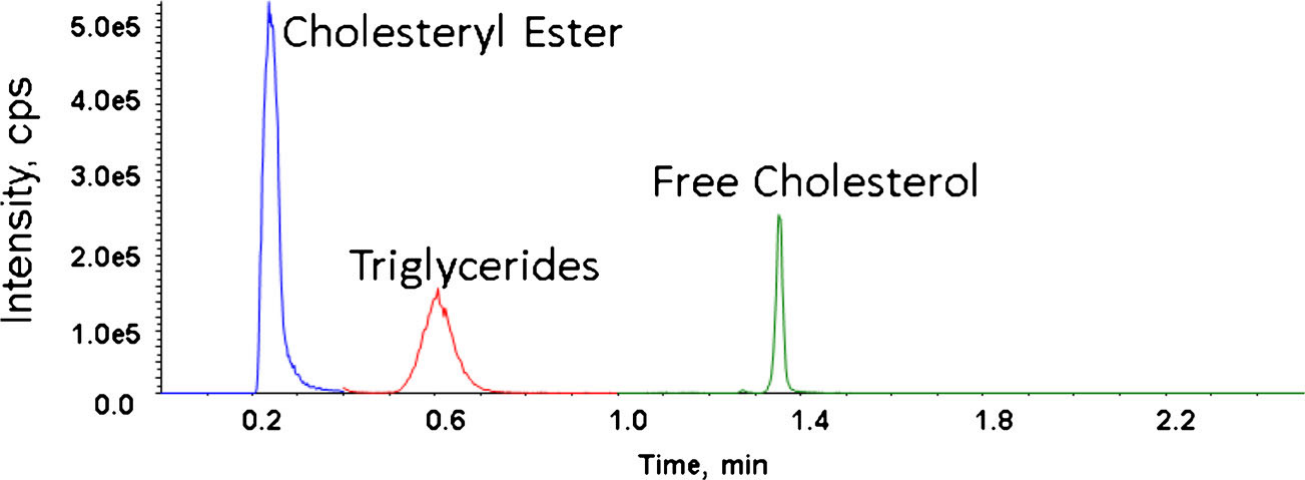
Analytical separation with gradient UHPLC

We explored isocratic HPLC, gradient HPLC, and gradient UHPLC separations. A typical chromatogram by injecting 1:100 diluted serum extracts is shown in Figure 1. In isocratic HPLC mode, the TG peaks had co-eluting interferences. Therefore, gradient HPLC was used, which improved the chromatographic resolution and separated TG from its interferences (data not shown). Gradient UHPLC was used for the final method (Figure 1) because it further improved chromatographic resolution, allowed for time segmented MS acquisition, and shortened the run time to 2.5 min/sample, allowing for the analysis of up to 192 samples per instrument per day on a typical analysis day.

The selected Kinetex UHPLC column was designed for HILIC mode separation. However, we used it without water in the eluent, in the normal phase mode by definition. EC and TG analytes were separated based on polarity as a lipid class without resolution by fatty acid ester species (FC does not have multiple ester species).

#### MS/MS Optimization for Free and Esterified Cholesterol

Although at different retention times both FC and EC species readily fragmented to *m*/*z* 369 ions, representing [M + H – H_2_O]^+^ and [M + H – CO_2_R]^+^ ions, respectively. Q1 spectra of FC, cholesteryl palmitate (16:0), cholesteryl oleate (18:1), and cholesteryl linoleate (18:2) are given in Supplementary Figure S2. The three EC species that are also the main endogenous ECs in typical human plasma were prepared at equal concentrations. The ion counts in the product ion spectra showed that all three ECs produced Q1 [M + H – CO_2_R]^+^ ion fragments with similar counts. LC-MS/MS analysis of the equal concentration solutions also showed similar response ratios for all three ECs (Supplementary Figure S3). Cholesteryl arachidonate, also included in the comparison of response ratios, showed a response ratio most similar to cholesteryl palmitate. Therefore, we concluded that any one of them alone was applicable as a calibration standard for all EC major species in serum. Because optimized DP resulted in a nonlinear calibration function for EC and FC due to detector saturation, the DP was reduced to non-optimum values, giving reduced signal response but linear calibration curves.

#### MS/MS Optimization for Triglycerides

Endogenous TG content in plasma is a mixture of TG analogs with fatty acyl moieties of various double bond position and carbon chain length. The key to the detection of all TG species with common MRM scans was the realization that at high DP of 262 V, much higher than typically used for organic compounds, all native TG species produce *m*/*z* 30–150 in-source fragments, and some of these fragments are common to all TG species regardless of acyl carbon chain length or number/position of double bounds. To identify these common TG fragments, we performed a series of experiments described below.

First, the formation of the low-mass in-source fragments from TG species was confirmed by infusing diluted corn oil, a mixture of triglycerides of biological origin. In single quad MS mode (Q1) at DP 50 V (without in-source CID), corn oil gave a series of triglyceride [M + H]^+^ molecular ions between *m*/*z* 830 and 900, and [M – CO_2_R]^+^ diglyceride fragments between *m*/*z* 550 and 750 (Supplementary Figure S4). The mass assignments of these molecular and fragment ions are described in the literature [10].

**Figure 2:**
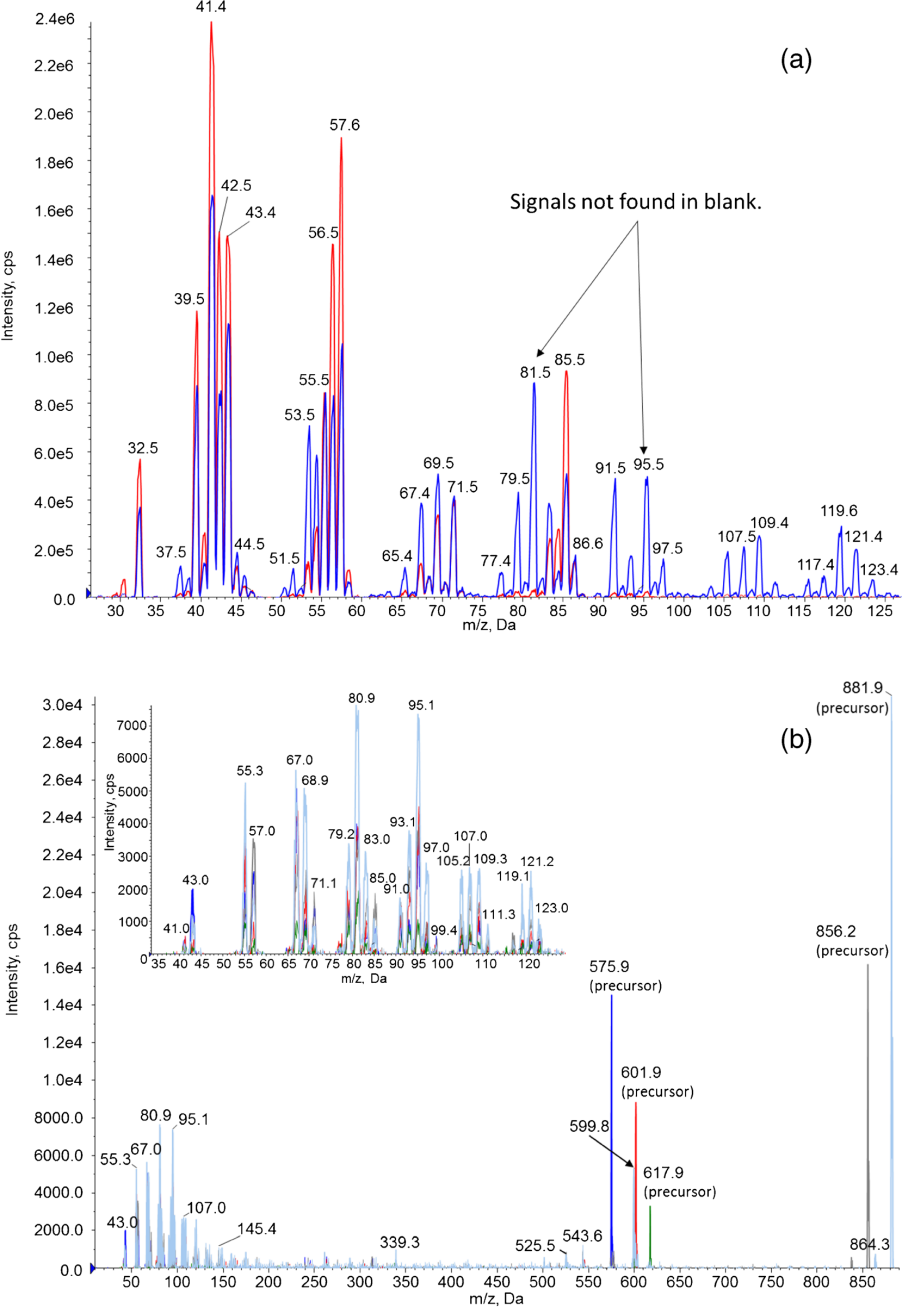
(**a**) Overlay of low mass fragment ions region generated by in source CID at DP of 200–300 V; corn oil (blue) and solvent blank (red). (**b**) Overlaid product ion scans from di- and triglyceride precursors, collected with DP 50 V and CE ramped from 5 to 130 V. Inset: expanded *m*/*z* 30–130 region. Precursor masses 881.9 (light blue), 856.2 (gray), 617.9 (green), 601.9 (red), 575.9 (dark blue). An expanded version of this figure is presented in Supplementary Figure S5

When the DP was raised above 200 V to achieve in source CID, several <*m*/*z* 150 fragment ions appeared from corn oil that were not found for the solvent blank. A similar pattern of low-mass product ions could also be observed in product ion scanning mode, when the diglyceride and triglyceride precursor ions were produced in Q1 using high CE ~80 V and their fragments detected in Q3, confirming their TG origin (Figure 2, right, insert). Based on their mass, the low mass fragment ions appeared to be a series of alkyl and alkenyl carbocations. We did not find published LC-MS or LC-MS/MS spectra of triglycerides that showed TG fragments below *m*/*z* 150, and their origin from fatty acid esters with a glycerol backbone has not been previously reported. Therefore, we had to perform further experiments to identify these low mass fragments and find those that are common to all TG species regardless of acyl chain length or double bond number/position.

**Figure 3:**
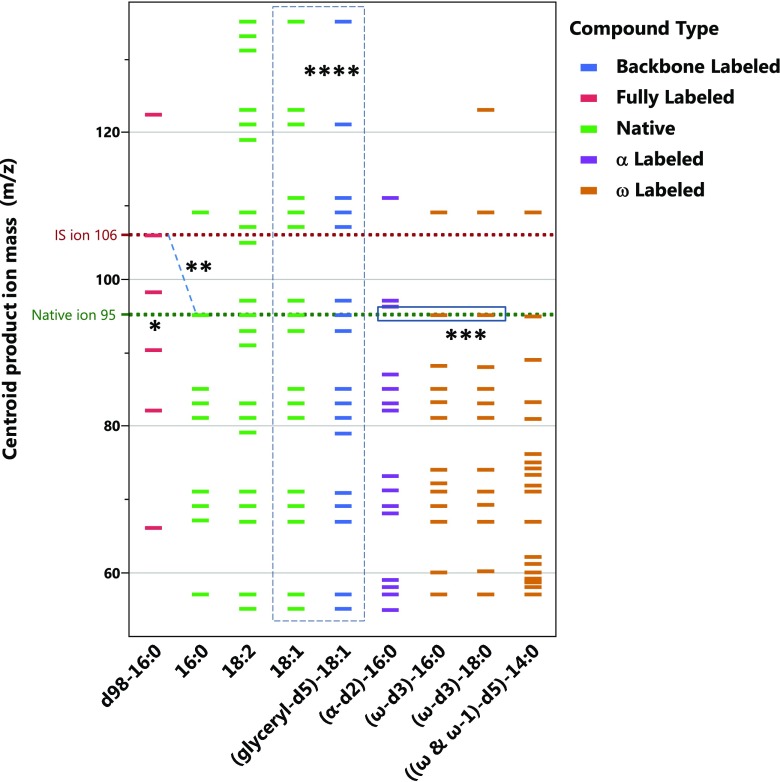
Graphical comparison of product ions detected above 10% of maximum intensity for different native and labeled TG species. *: Dotted line indicating product ions that were the best candidates to be used as MRM precursors for quantification. **: Native ion C_7_H_11_ with *m*/*z* 95 correspond with fully labeled ion C_7_D_11_ with *m*/*z* 106. ***: Absence versus presence of the C_7_H_11_ *m*/*z* 95 ion for α-carbon versus the ω-carbon labeled species, indicating that the *m*/*z* 95 ion contains the α-hydrogens. ****: Identical *m*/*z* ions (in dashed rectangle) from native and glyceryl-d_5_-labeled 18:1 species, indicating that the low mass fragments do not originate from the glycerol backbone

Next, to identify common TG class-specific low-mass fragments (common to TGs as a lipid class instead of TG species of different fatty acid derivatives), we infused pure TG compounds and their stable isotope labeled analogs in product ion scanning mode (MS2) using DP 50 V and high CE, between 50 and 130 V. The *m*/*z* 50–130 region of the product ion spectra for each TG species are shown in Supporting Information. A graphical comparison of *m*/*z* masses of the main fragment ions from various native and stable isotope labeled TGs is shown in Figure 3.

Of the native species, the fully saturated tripalmitin (16:0) spectrum showed a series of alkyl and alkenyl carbocations, from *m*/*z* 43 (C_3_H_7_) to *m*/*z* 137 (C_10_H_17_). The spectra of monounsaturated triolein (18:1) and polyunsaturated trilinolein (18:2) showed numerous additional product ions, assignable to hydrocarbons with varying degrees of unsaturation. Some product ions (i.e., *m*/*z* 91 (C_7_H_7_) and *m*/*z* 159 (C_12_H_15_) and numerous others) were observed only for trilinolein (18:2). However, several product ions, such as *m*/*z* 67, 81, 85, 95, and 109, were common to all the above native TG species (Figure 3 and Supplementary Figures S8–S10).

Individual pure native and labeled TG compounds were then infused to identify the carbon chain position-origin of the low mass product ions. The low mass product ion spectrum of glyceryl-d_5_-triolein (18:1), which is deuterated only on the glycerol backbone, was nearly identical to native triolein (18:1) indicating that the low mass products did not contain the glycerol backbone, and originate from the fatty acid carbon chains (Supplementary Figure S11). Conversely, in the product ion spectrum of the d_98_-tripalmitin (16:0), where every hydrogen has been substituted with a deuterium, the pattern of deuterated product ions could be assigned to the same alkyl and alkenyl ions as the native tripalmitin, mass shifted in accordance with its number of deuteriums. These assignments corresponded with non-deuterated ions of native tripalmitin (16:0), confirming that the low-mass ions come from the fatty acid moieties (Supplementary Figure S12).

We also examined one α-carbon labeled (16:0, d_2_), two ω-carbon labeled (16:0 and 18:0, d_3_), and one ω/(ω-1)-carbon (14:0, d_5_) labeled triglyceride species that had three identical saturated fatty acid moieties (Supplementary Figures S13–S16). For the α- and ω-carbon labeled species, we observed a series of 1–2 u or 1–3 u shifted product ion masses, respectively. When the deuterium label was both on the ω and (ω-1) carbon, we saw product ions shifted 1–5 u. These shifts indicate complex fragmentation pathways most likely through cyclic and alkenyl intermediates involving the carbons on the two ends of the carbon chains. By comparing these labeled species we were able to sort out the α-, ω- as well as non-α- and non-ω-origin of numerous product ions.

On the basis of the fragments observed from the native TGs, and the d_98_-tripalmitin, one might expect the α- and ω-labeled saturated TGs to show the same diversity of fragments as the native 16:0 TG. However, a comparison of native 16:0 versus the partially labeled saturated TGs in Figure 3 shows a greater diversity of fragments for the partially labeled TGs. For example, in Figure 3, native 16:0-TG has nine ions meeting the intensity threshold, whereas (α-d_2_)-16:0 has 15 ions, (ω-d_3_)-16:0 has 13 ions, (ω-d_3_)-18:0 has 13 ions, and [(ω & ω-1)-d_5_]-14:0 has 19 ions. We explain this difference as being attributable to many of the low mass fragments originating from multiple sources. Of the 15 ions featured in Table 2, only four contain the α-hydrogens exclusive of the ω-hydrogens, none contain ω-hydrogens exclusively, and four contain (at least) both α- and ω-hydrogens of the fatty acids. From the labeling of only α- or ω-hydrogens, we have also been able to assign five of the ions in Table 2 as containing neither the α- nor ω-hydrogens. We can also tentatively claim that some of the ions in the aforementioned group contain ions from (ω-1)-hydrogens, given the great diversity of fragments observed in the last column of Fig. 3.  Although we cannot rule out the impact of carbon chain length, since the only TG labeled in the (ω-1) position contains 14 carbons, rather than 16 or 18.

**Table 2:** Assignment of Low-*m*/*z* Product Ions Based on Mass and Origin of Hydrogens by Comparison of Native and Deuterium Labeled Species in Figure 3

Product ion (*m*/*z*)	Assignment	Origin of hydrogens from position on fatty acid
43	C_3_H_7_	α and ω
55	C_4_H_7_	Neither α nor ω
57	C_4_H_9_	α and ω
67	C_5_H_7_	α
69	C_5_H_9_	Neither α nor ω
71 (82)	C_5_H_11_ (C_5_D_11_)	α and ω
81	C_6_H_9_	Not assigned
83	C_6_H_11_	Not assigned
85 (98)	C_6_H_13_ (C_6_D_13_)	α and ω
95 (106)	C_7_H_11_ (C_7_D_11_)	α
97	C_7_H_13_	Neither α nor ω
99	C_7_H_15_	Neither α nor ω
109 (122)	C_8_H_13_ (C_8_D_13_)	α
123	C_9_H_15_	α
137	C_10_H_17_	Neither α nor ω

In spite of the different carbon chain length and labeling position, the product ion spectra of the labeled TGs showed several common ions with the native species. Most importantly, the native tripalmitin showed a common C_7_H_11_ product at *m*/*z* 95 with the ω-labeled but not with the α-labeled analogous 16:0 species. This information confirmed that the C_7_H_11_ *m*/*z* 95 ion contained hydrogens originating from the α-carbon, and not the ω-carbon. The mass assignments of the low mass ions with identifiable origins are summarized in Table 2.

The ions in the product ion spectra at high CE also occurred as in-source CID fragments in the Q1 spectra using DP > 200 V. The similarities of these spectra are shown with corn oil (Figure 2) and pure tripalmitin, triolein, and trilinolein (Supplementary Figures S17–S19). Many of these CID fragments had interferences as seen in a solvent blank; however, there were some that did not show interfering ions. Based on the product ion spectra of these interference-free CID fragments (Supplementary Figure S20), the *m*/*z* 95/67 and 95/55 transitions were chosen for greatest intensity. Similarly, for d_98_-tripalmitin (used as IS), *m*/*z* 98/50 and 106/74 transitions were chosen. The transition 98/50 of d_98_-tripalmitin is analogous to 85/43 of the native triglyceride, the latter of which was not used because of the precursor background which was exhibited in Figure 2a. The transition 106/74 of d_98_-tripalmitin is analogous to 95/67 of the native triglyceride.

To compare the fragmentation efficiency of TG species, individual solutions of tripalmitin (16:0), triolein (18:1), and trilinolein (18:2) were prepared at equal concentrations and we measured their LC-MS/MS response ratios relative to d_98_-triparmitin (IS for TG) (Supplementary Figure S21). The response ratios of triolein and tripalmitin were 30% and 5% of trilinolein, respectively. We also compared the response ratios measured from eight serum samples after adjusting for the differences in the Total-TG concentrations that were determined using a GC-MS based method by the Lipid Standardization Laboratory of CDC. After adjustment for the serum concentration, the response ratios for the eight serum samples varied only ±3%. Therefore, we concluded that TG species in serum ionize with similar average efficiency. Nevertheless, we prepared a representative mix of the three pure TGs, in a 514:313:173 ratio established based on literature data [11], to be used as external calibrators.

### Limit of Detection and Linear Quantification Range

The lower limit of detection (LOD), using 50 μL aliquots of AF4 fractions or 1:100 diluted serum, was determined based on three times the extrapolated standard deviation at zero concentration (3S_0_). At the LOD, the concentration in the sample well was 0.04 mg/dL for EC (expressed as the free cholesterol mass equivalent), 0.01 mg/dL for FC, and 0.05 mg/dL for TG. The lower limit of quantification (LLOQ) was determined based on lowest calibration standard concentration with 20% deviation of calculated values, and upper limit of quantification (ULOQ) was determined by the highest calibration standard concentration with 15% deviation of calculated values [12]. LOD, LLOQ, and ULOQ values are summarized in Table 3. The LOD and LLOQ were limited by the injection volume and the diluted sample aliquot volume used for extraction. The maximum injection volume that did not degrade the chromatographic separation was 6 μL.

**Table 3:** Limits of Detection (LOD) in 50 μL Aliquots of Size Fractions or 1:100 Diluted Serum, Lower Limits of Quantification (LLOQ), and Upper Limits of Quantification (ULOQ) in Serum

Analyte	LOD in 50 μL diluted aliquots (mg/dL)	LLOQ concentration in serum (mg/dL)	ULOQ concentration in serum (mg/dL)
Esterified cholesterol	0.04	20	397
Free cholesterol	0.01	15	132
Triglycerides	0.05	30	530

The LLOQ-ULOQ calibration range was applicable to the analysis of both whole serum samples and size fractions collected by AF4. The concentrations in the AF4 size fractions were the equivalent of approximately 1:50 to 1:2000 serum dilutions. For 1:100 diluted whole serum, the LLOQ was well below the physiologically relevant range. At the ULOQ, the serum concentrations (before dilution) were 397 mg/dL for EC, 132 mg/dL for FC, 529.9 mg/dL for Total-C, and 509 mg/dL for Total-TG, allowing the quantification of most human samples. The sample dilution could be adjusted to achieve a higher effective ULOQ if desired.

### Accuracy and Bias for Total-C and Total-TG Relative to Reference Materials

Using the external calibration series prepared in-house from a representative mix of purified materials, two NIST and five LSP reference materials were measured on five days. NIST-SRM-1951c Level 1 had certified values of 152.44 mg/dL for total cholesterol and 152.0 mg/dL for total glycerides. Level 2 had certified values of 241.41 mg/dL for total cholesterol and 145.4 mg/dL for total glycerides. Relative to the NIST assigned values, the bias in our measurements versus the certified concentrations of the SRMs was –4.2% for Total-TG and –10.2% for Total-C (calculated as sum of FC and EC). Relative to the CDC assigned values (using GC/MS), the average bias in our measurements versus the certified concentrations of the five LSP standards was –3 ± 5% for Total-TG and –10 ± 4% for Total-C. Although the biases were higher than acceptable for clinical measurements, they were highly reproducible. Because of the consistency of the bias, our method can be harmonized by applying a corresponding correction factor based on either NIST or CDC standards.

### Calibration with Value Assigned Serum

An alternative way to achieve harmonization is by using the NIST SRM 1951c serum [13] as a calibrator. For this purpose, the FC and EC content of the SRM 1951c serum had to be determined because NIST only assigns values for total cholesterol (sum of FC and EC). Using pure FC external calibrators, for Level 1 and Level 2 SRM-1951c (diluted 1:100 in five replicates) contained FC = 36.4 mg/dL and 52.6 mg/dL, and EC = 116.0 mg/dL and 188.8 mg/dL, calculated as EC = Total-C – FC. Furthermore, the NIST certified total glycerol content of the SRMs 1951c samples also includes free glycerol. However, the method presented here measures only TG esters without glycerol. Because most serum samples contain only ~3% glycerol, the value assignment of the SRM 1951c sera was used without correction.

Using Level 1 SRM 1951c serum as calibrator, an eight point calibration series was prepared, 0.04–4.0 mg/dL for EC, 0.01–1.3 mg/dL for FC, and 0.05–5.3 mg/dL for TG. Using this dilution series, five LSP reference materials were measured; diluted 1:100 in five replicates and each dilution replicate was analyzed three times (n = 15). As expected, the average bias relative to the assigned values were much less than those obtained with pure external calibrators, +2.1% for TC and +3.7% for TG. The average CV was 3.1% for TC and 4.2% for TG. The Cholesterol Reference Method Laboratory Network (CRMLN) laboratories are required to have a secondary reference method that has an average bias ≤1% and CV ≤1% for total cholesterol and bias ≤2.55% and CV ≤3.95% for total glycerides. Colorimetric methods performed on clinical analyzer platforms are typically required to have bias ≤3% and CV ≤3% for total cholesterol and bias ≤5% and CV ≤5% for total glycerides relative to a standard reference materials (SRM) value assigned by a CRLMN laboratories.

It should be noted that the accuracy of the reported triglyceride value depends on the ratio of fatty acids in the TG not deviating substantially from the ratio found in the calibrators, whether the calibrators are prepared from value-assigned serum or mixtures of purified triglycerides. For example, a markedly high percentage of linoleate relative to oleate would cause an overestimation of TG content.

### Reproducibility

Reproducibility was assessed with calibrators prepared from pure FC, CE, and TG species, and also from dilution of Level 1 SRM 1951c serum. Using representative mix of FC, CE, and TG as calibrator, repeated analysis of the in-house prepared quality control pool showed coefficient of variation (CV) intra-day (n = 2) and inter-day (n = 40) of 5% and 5.8% for FC, 5.2% and 8.5% for Total-EC, and 4.1% and 7.7% for Total-TG, respectively. Using dilution series of Level 1 SRM 1951c serum as calibrator, the inter-day CV (n = 27) was 3.4% for TC and 5.6% for TG.

### Application to Total-C and Total-TG Measurements in Patient Samples

**Figure 4:**
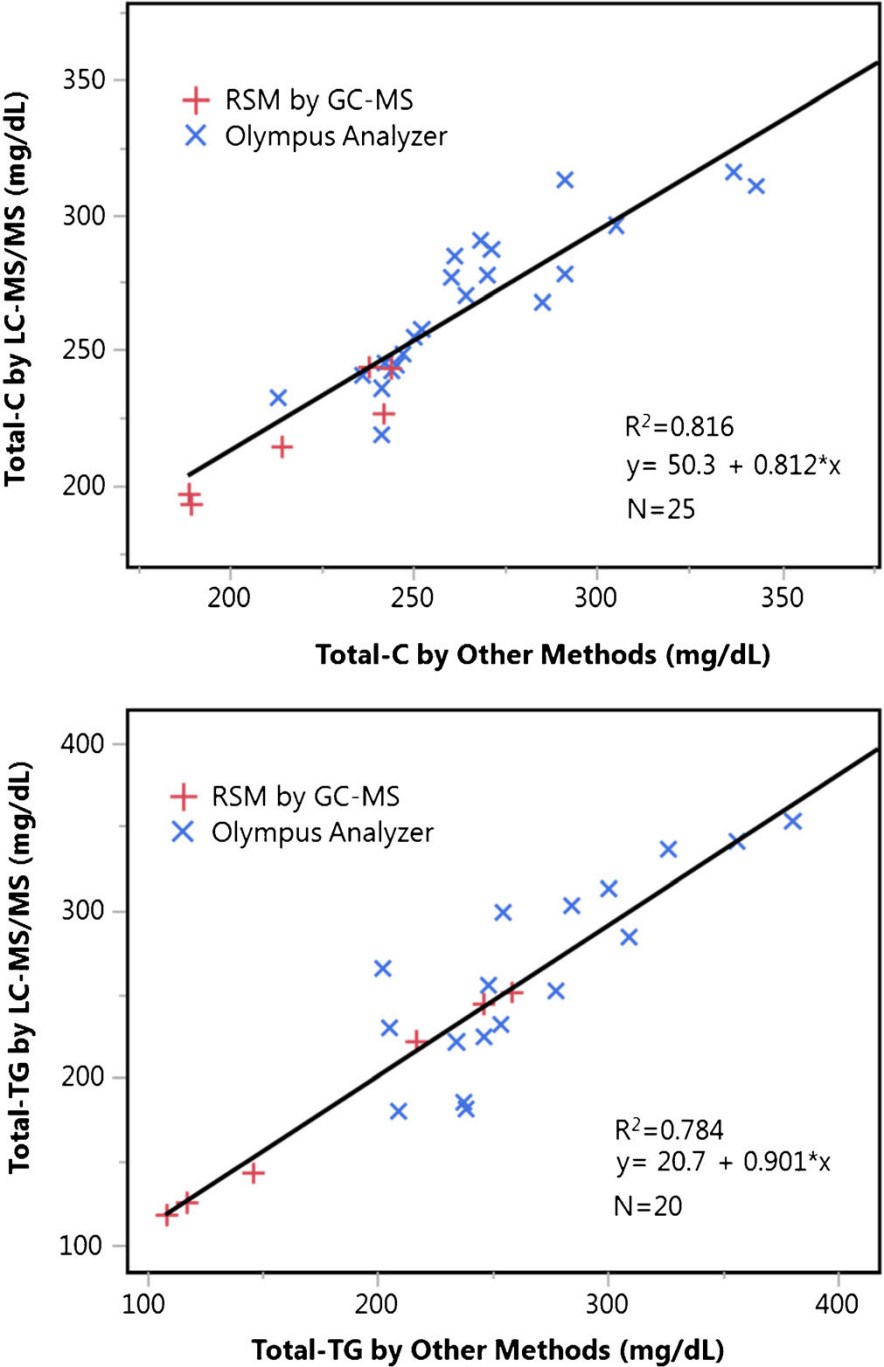
Comparison of Total-C and Total-TG concentrations (mg/dL) measured by LC-MS/MS in this work and values obtained by Olympus clinical analyzer and by GC-MS reference method. The linear regressions utilize all data points

Thirty hyperlipidemic patient samples were analyzed with our LC-MS/MS method using NIST certified serum as a calibrator. The samples were also analyzed in an unidentified clinical laboratory that used an Olympus clinical analyzer. In Figure 4, the LC-MS/MS and Olympus results are compared along with the serum reference material results. The differences were 2.84 ± 6.98% for Total-C and –1.16 ± 14.33% for TG. Although the average biases are not large, the slopes and intercepts of the correlations predict a positive bias at the low end of the biologically relevant range, and a negative bias at the high end. This method should be used with caution in whole samples with extremely high or low values, until comparisons to clinical methods can be made at extreme levels.

### Application to Measurements in Size Fractions

Owing to high sensitivity and throughput, our LC-MS/MS method offers the greatest advantage for the analysis of lipoprotein sub-fractions in this work generated based on hydrodynamic size by AF4 using 50 μL serum. To preserve the resolution allowed by AF4 (half peak width of individual species ~1 nm), the lipoproteins were collected in 40 fractions. The sample preparation was performed in 96-well plate format. Each plate contained an eight level calibrator series, a blank, three replicate dilutions of a QC serum pool, four 1:100 parallel dilutions of the unknown whole serum samples, and 80 corresponding unknown AF4 fractions.

**Figure 5:**
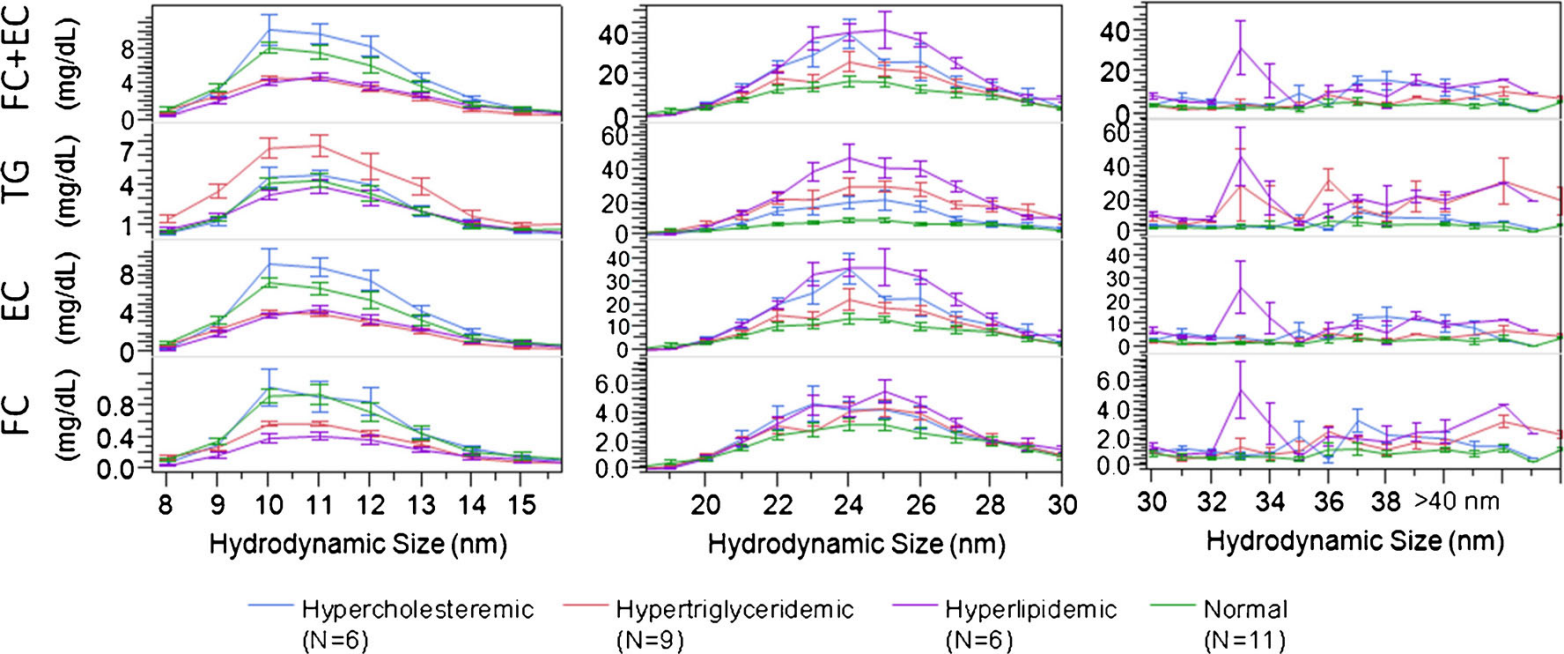
Overlay of average lipid profiles versus hydrodynamic size for normal, and three types of dyslipidemic patient groups (error bars represent standard errors about the group mean). No size measurement could be made above 40 nm

The UHPLC-CID-MRM method allowed the FC, EC, and TG analysis of all 40 fractions per sample in <3 h (including extraction time), along with triplicate total serum measurements using only an additional 1.5 μL serum. After the instrument run, FC, EC, and TG concentration versus size profiles were generated. The concentrations in the fractions were converted into sub-species concentrations in serum, treating each size fraction as a sub-species. As an example, Figure 5 shows profiles in overlay as averaged for normal and three types of dyslipidemic patient groups (error bars represent standard errors about the group mean).

Our data allowed us to show significant differences among dyslipidemic groups in spite of the limited number of donors in each group. Coupling size and lipid analysis allows the calculation of other lipoprotein characteristics of lipoprotein sub-fractions, such as FC/(EC+TG) representing the FC content of the surface relative to the core lipids, or TG/EC ratio of the core (Supplementary Figure S22). These particle characteristics are indicative of underlying lipid metabolism pathways, i.e., cholesterol efflux, FC to EC esterification and TG hydrolysis rates. Of note, the specificity, sensitivity, and simplicity, as well as high-throughput and multiplexed nature of our method enabled us to obtain fraction information from <100 μL serum with 500 fraction/d throughput. Furthermore, to obtain the FC, EC, and TG levels required the use of only 50 μL from the collected 250 μL fractions, leaving opportunity for additional apolipoprotein and phospholipid analyses, which we plan to report in a future publication.

The reproducibility of our LC-MS/MS method is comparable with colorimetric assays used in common clinical settings. The accuracy of the colorimetric assays (<3% CV for TC, <5% CV for TG) is usually characterized in a narrow concentration range. Similar to colorimetric assays, our LC-MS/MS sample preparation can be performed in 96-well plate format, in three steps without sample transfer, using as little as 0.5 μL serum and one LC-MS/MS injection per sample to calculate serum concentrations of FC, EC, and TG (without contribution from free glycerol). It should be noted that we typically pipette 10 μL serum and dilute it 1:100 for use across multiple assays, because of limitations in pipetting accuracy at smaller volumes. To acquire the same information by colorimetric assays would have required 60–120 μL serum (on a clinical analyzer platform typically more), two (or four) sample preparations per unknown, and at least four absorbance readings per sample. Indeed, LC-MS/MS requires investment in sophisticated instrumentation. On the other hand, colorimetric assays rely on an infrastructure of reagent suppliers (four different enzymes for TG and two enzymes for EC analysis), and application in high-throughput and automated fashion requires investment in chemical analyzer platforms.

## Conclusions

The main contribution of this work is the demonstration of the applicability of LC-MS/MS for high-throughput simultaneous quantification of nonpolar lipids in serum using APCI ionization with in-source CID fragmentation without the need for ester hydrolysis. We have also reported the observation of low-mass fragment ions of TGs, generated by both MS/MS and in-source CID, some of which appear to be common to TGs as a class. By chromatographic separation and using generic fragments for MRM-MS detection, FC along with EC and TG species as lipid classes could be determined. The method allows accurate and reproducible quantification in diluted serum and in lipoprotein sub-fractions. This LC-MS based approach is highly applicable for research studies where samples are substantially diluted or the amount of archived samples are limited.

## Electronic supplementary material

Below is the link to the electronic supplementary material.

ESM 1 (8.60 MB)
