# Supplementary material for: Simultaneous Quantification of Free Cholesterol, Cholesteryl Esters, and Triglycerides without Ester Hydrolysis by UHPLC Separation and In-Source Collision Induced Dissociation Coupled MS/MS
Source: J Am Soc Mass Spectrom. 2017 Aug 11;28(11):2319–29. doi: 10.1007/s13361-017-1756-2 (PMC5645443; doi:10.1007/s13361-017-1756-2)

Supplementary Information

Journal of the American Society for Mass Spectrometry

Simultaneous Quantification of Free Cholesterol, Cholesteryl Esters and Triglycerides without Ester Hydrolysis by UHPLC Separation and In-Source Collision Induced Dissociation Coupled MS/MS

Michael S. Gardner; Lisa G. McWilliams; Jeffrey I Jones; Zsuzsanna Kuklenyik; James L. Pirkle; and John R. Barr

Division of Laboratory Sciences, National Center for Environmental Health, Centers for Disease Control and Prevention, Chamblee, GA, United States

Corresponding author: Dr. John R. Barr <jbb0@cdc.gov>

Size Fractionation of serum lipoproteins by Asymmetric flow field-flow fractionation (AF4):

This separation technique is based on the fundamental nature of laminar flow. A liquid medium passes through a thin channel which causes it to adopt a parabolic velocity profile across the height of the channel, with stream velocities faster at the center of the channel and slower near the walls. When serum samples are injected into this stream, contained particles are subjected to a second, perpendicular field created by withdrawal of carrier fluid through a semipermeable membrane that drives them toward the accumulation wall, where they experience slower flow rates. Once they are concentrated at the accumulation wall, particle species begin to diffuse up away from the accumulation wall via Brownian motion into higher velocity flow regimes. Differential retention is caused by the different average height achieved by particles of different sizes, with smaller particles having a higher average height which results in them eluting faster. The AF4 separation requires no filtration or other sample pretreatment and is achieved by gentle fluid dynamics; both of these elements minimize the risk of introducing artifacts during separation

Table S1 – Fatty acid (FA) specific MS/MS products observed at low collision energy potential (30 V).

Triglyceride	Precursor (m/z)	Product (m/z)	Neutral loss
16:0/16:0/16:0 (tripalmitin)	808.1	551.8	256: FA(16:0)
18:1/18:1/18:1 (triolein)	886.2	603.9	282: FA(18:1)
18:2/18:2/18:2 (trilinolein)	880.2	599.9	280: FA(18:2)
d98-(16:0/16:0/16:0)	906.7	619.0	d31-FA(16:0)
Glyceryl-d5-(18:1/18:1/18:1)	891.2	608.9	FA(18:1)
(α-d2)-16:0/(α-d2)-16:0/(α-d2)-16:0	814.1	555.9	d2-FA(16:0)
(ω-d3)-16:0/(ω-d3)-16:0/(ω-d3)-16:0	817.2	557.8	d3-FA(16:0)
(ω-d3)-18:0/(ω-d3)-18:0/(ω-d3)-18:0	901.3	613.8	d3-FA(18:0)
(13,13,14,14,14-d5)-14:0/(13,13,14,14,14-d5)-14:0/(13,13,14,14,14-d5)-14:0	739.0	505.8	d5-FA(14:0)

Figure S1. Comparison of measured concentrations of six randomly selected unknown samples prepared using nitrogen or air for sample evaporation. All other preparation and analysis conditions are held constant.

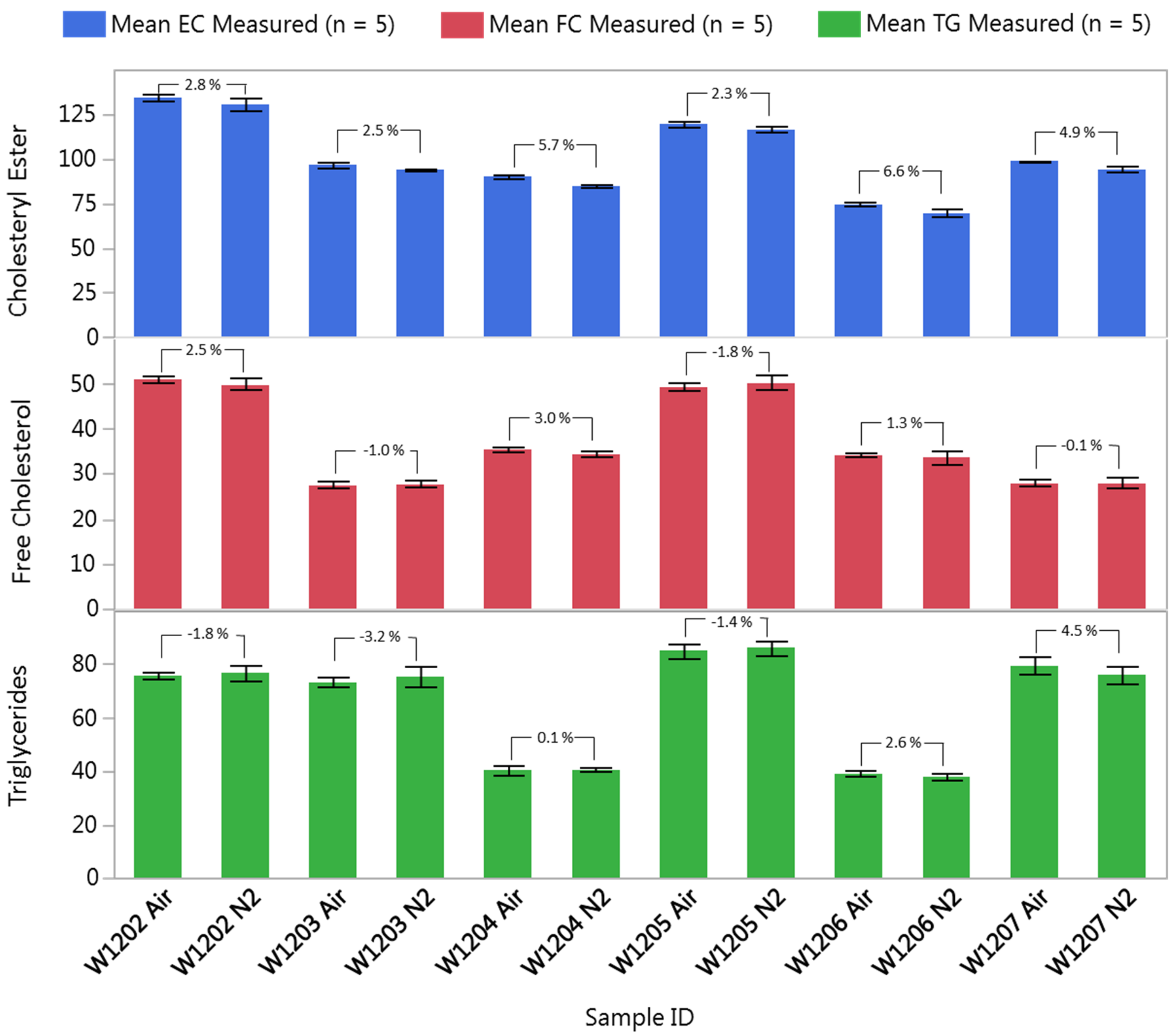

Figure S2. Blank subtracted Q1 spectra of (a) cholesterol, (b) cholesteryl palmitate, (c) cholesteryl oleate, and (d) cholesteryl linoleate. DP = 20 V. Data acquired by UHPLC-MS with the same chromatographic conditions as for UHPLC-MS/MS.

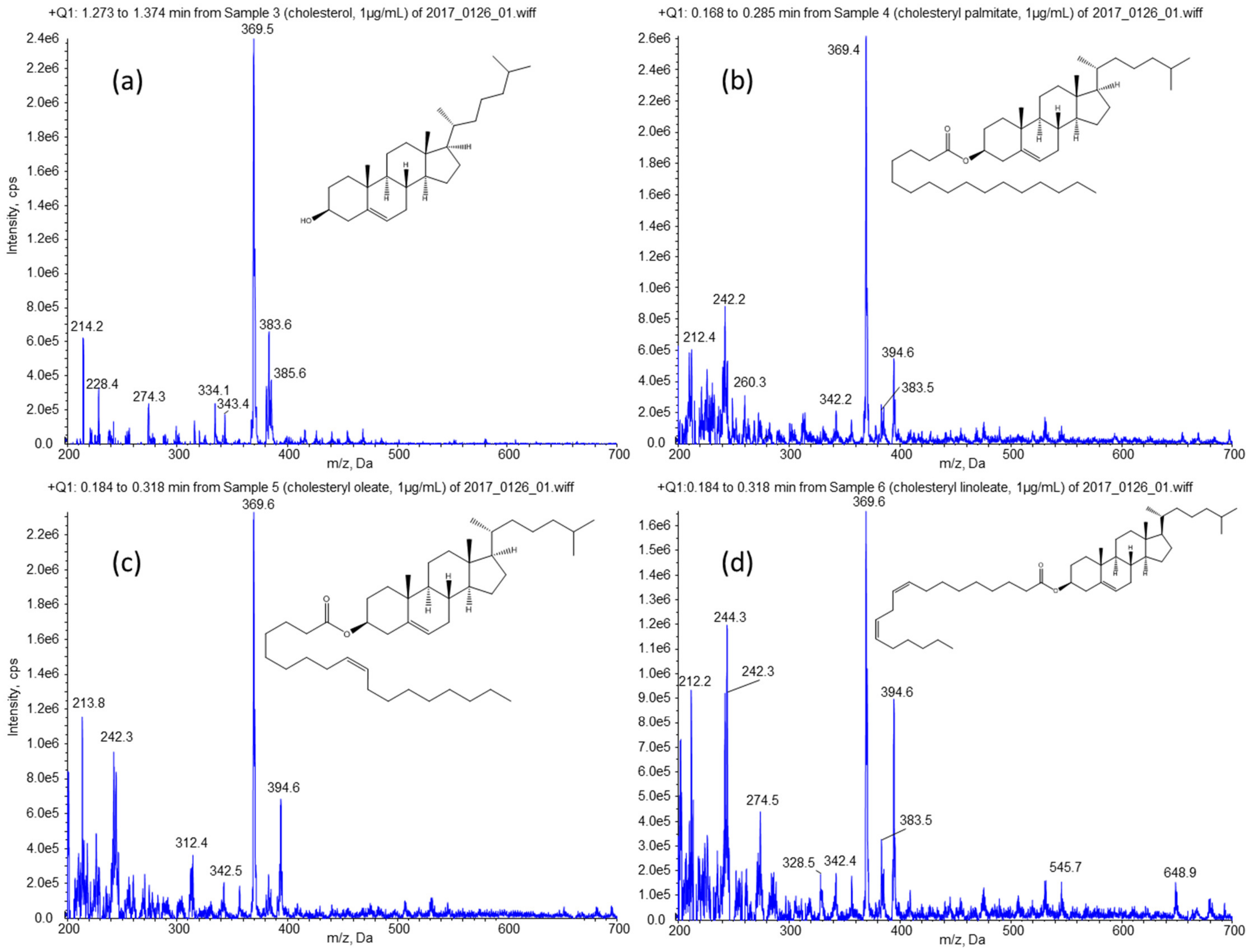

Figure S3: Comparison of response factors of EC showing similar efficiency of fragmentation monitoring the 369/161 m/z transition. Also, the response ratio from concentration assigned standard reference materials where the response ratios were normalized for the expected concentration, showing on average the response ratio of the different EC species present in the serum samples were similar. For serum samples with known TC values, FC was directly measured and expected EC was calculated by subtracting measured FC from known TC. Each error bar is constructed using one standard deviation from the mean.

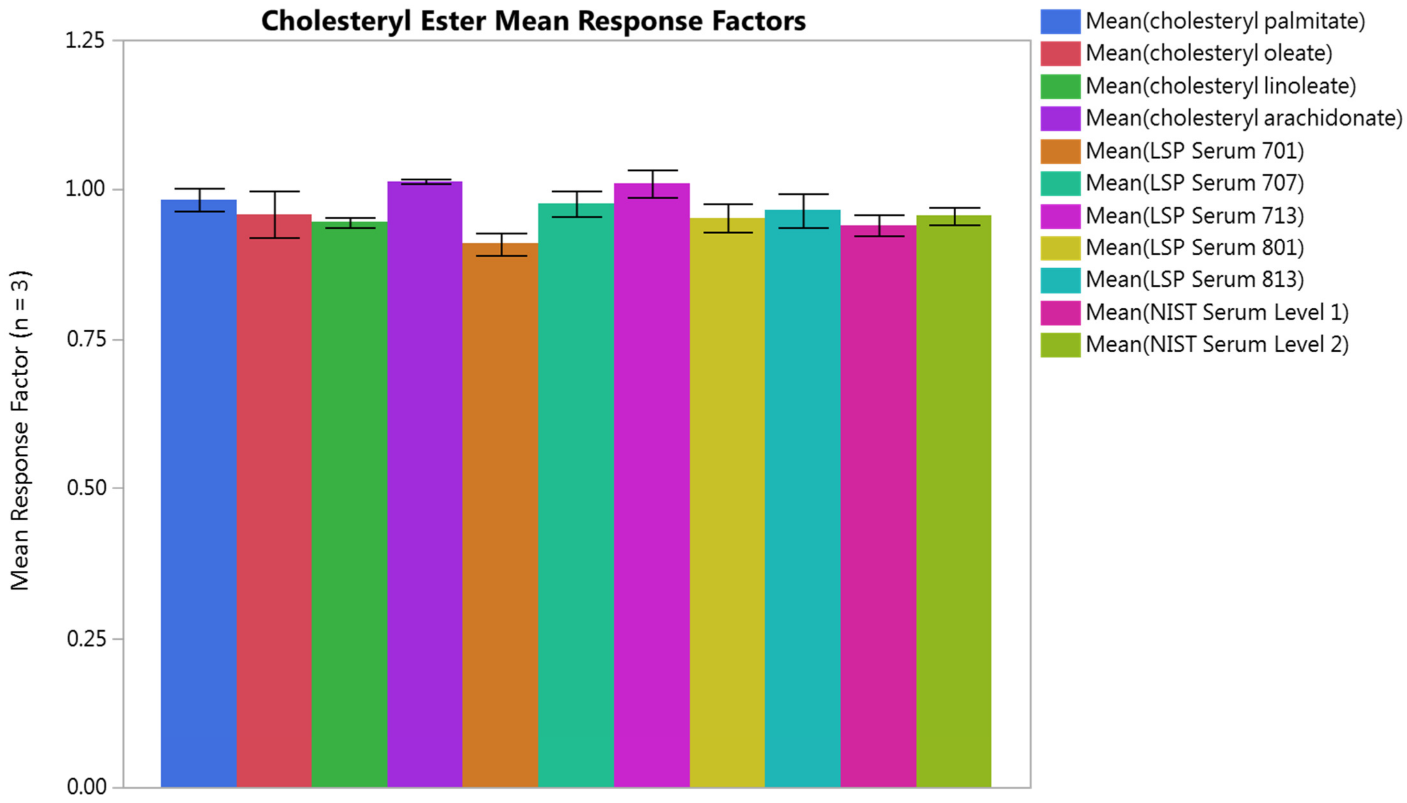

Figure S4. Blank subtracted corn oil Q1 spectrum. DP = 50 V.

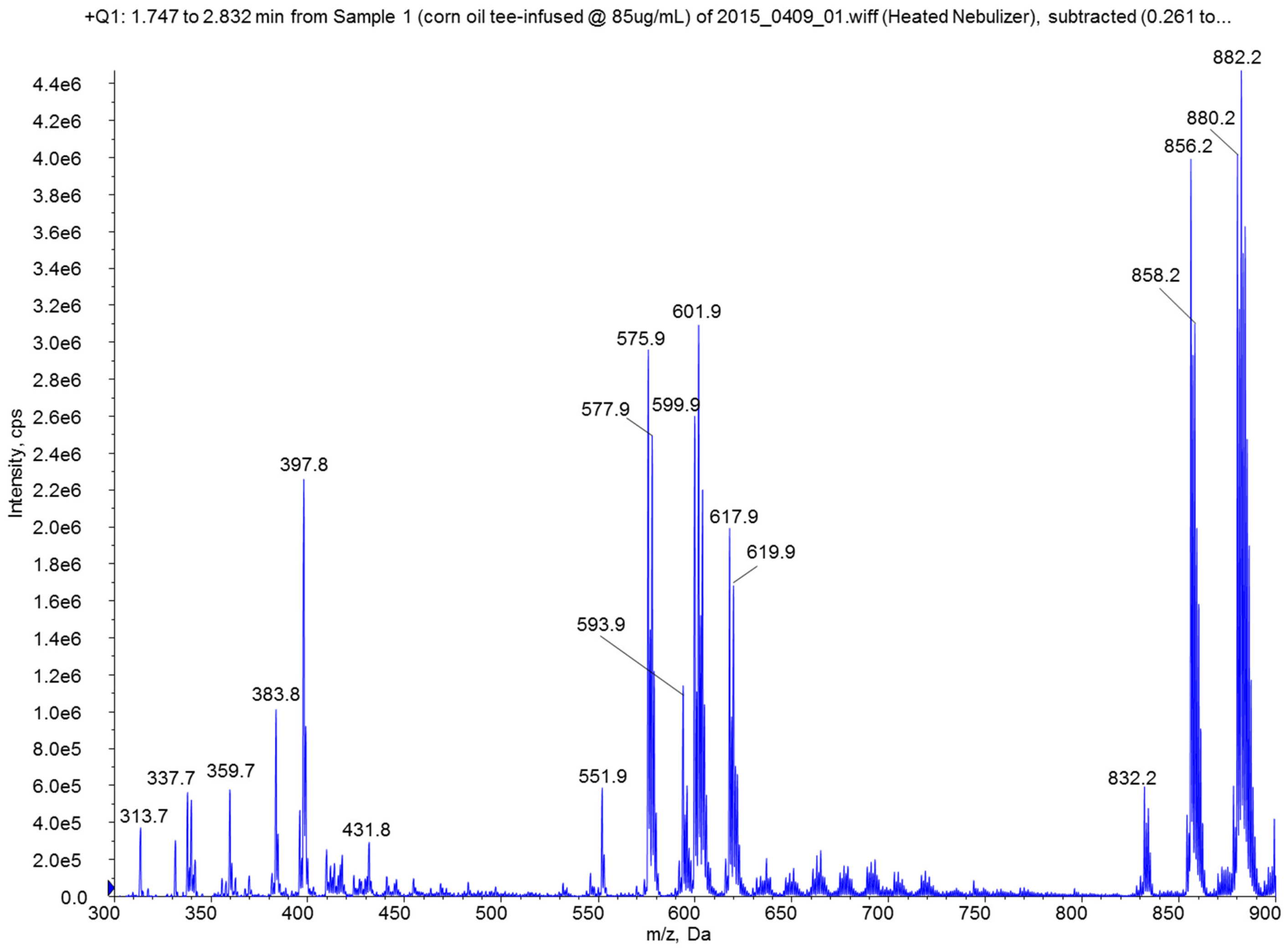

Figure S5. Product ion spectrum of corn oil. DP 50 V, CE of 5-130 V ramped. Expanded version of spectrum in Figure 2(b).

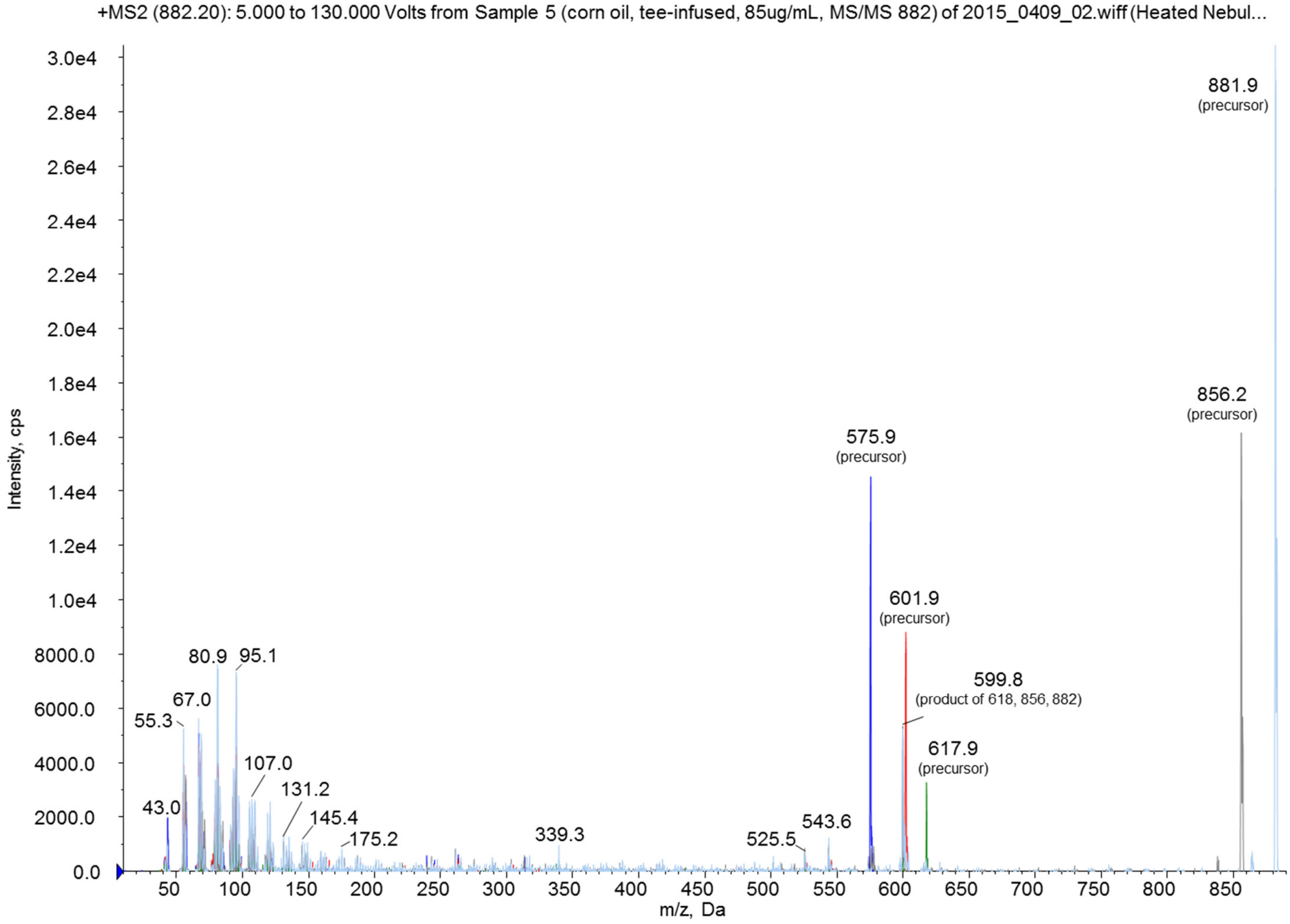

Figure S6. Product ion spectrum of corn oil. Low mass region in Figure S3. DP 50 V, CE of 5-130 V ramped. Expanded version of insert in Figure 2(b) inset.

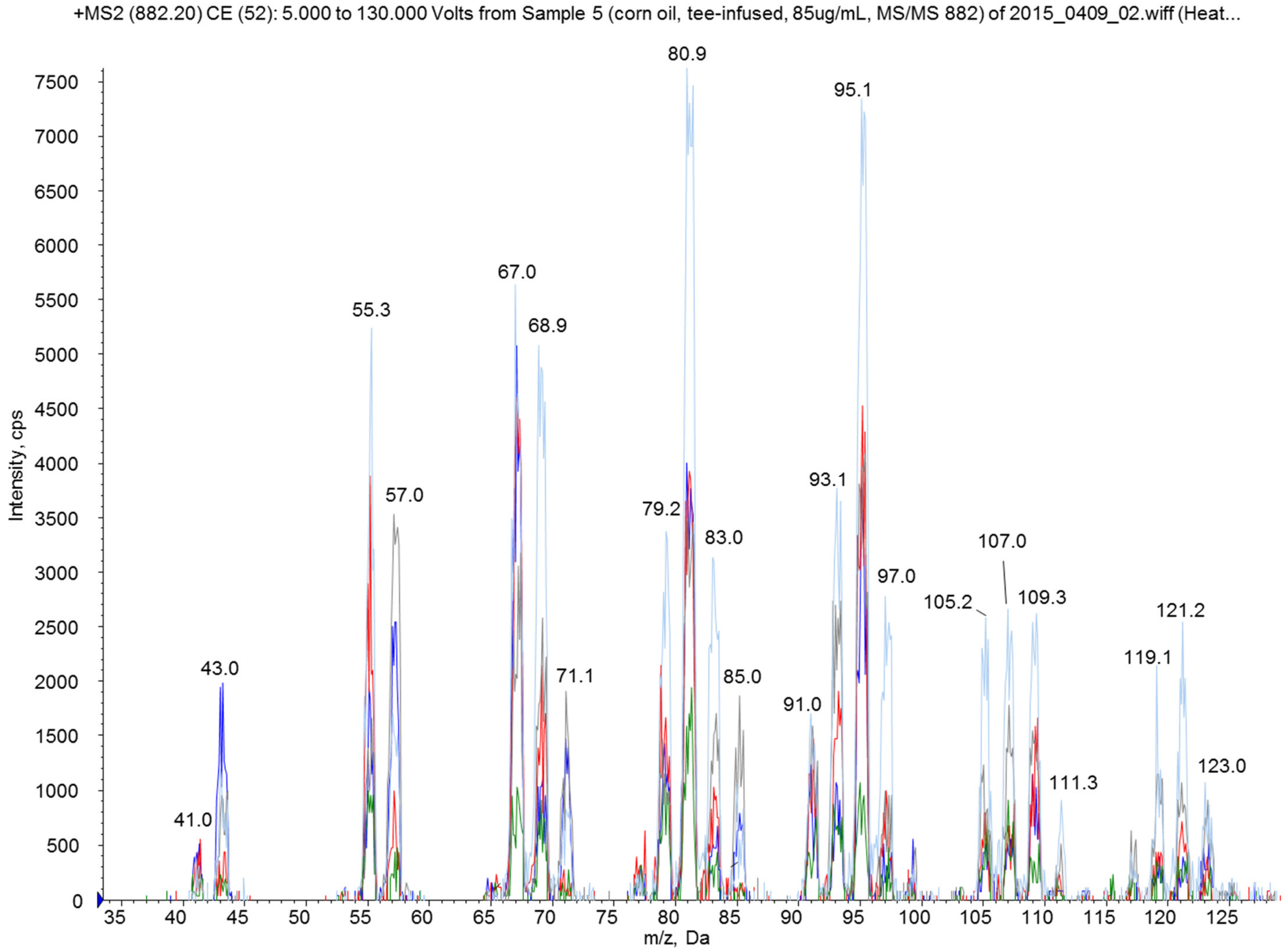

Figure S7. Overlay of single quad MS spectrum (Q1) from corn oil expanded at m/z 50-130; DP 300-400 V ramped average, in-source CID fragmentation. Expanded version of spectrum in Figure 2(a) in main text.

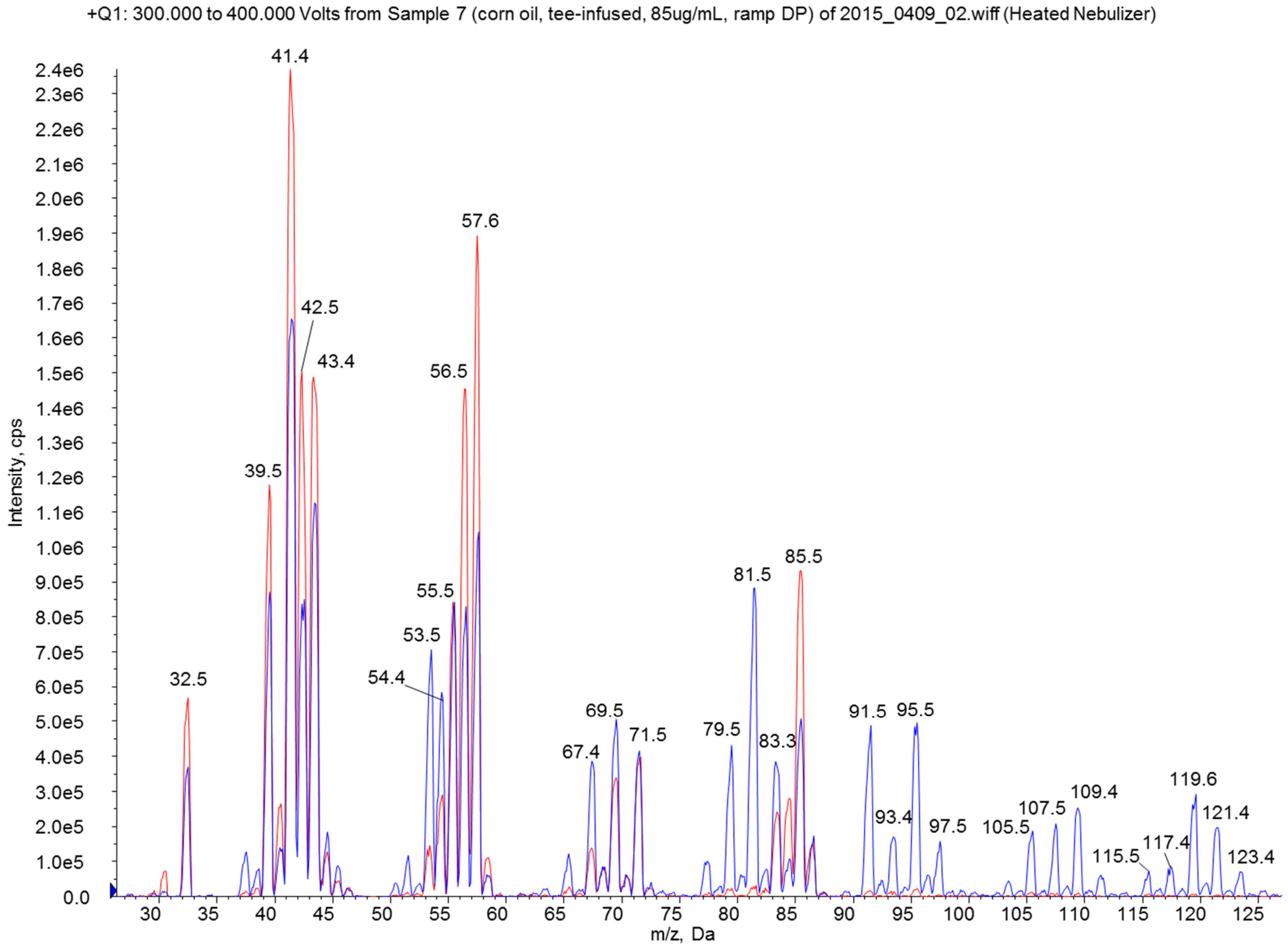

Figure S8. MS/MS of Tripalmitin at CE = 80V. Data summarized in Figure 3 of main text.

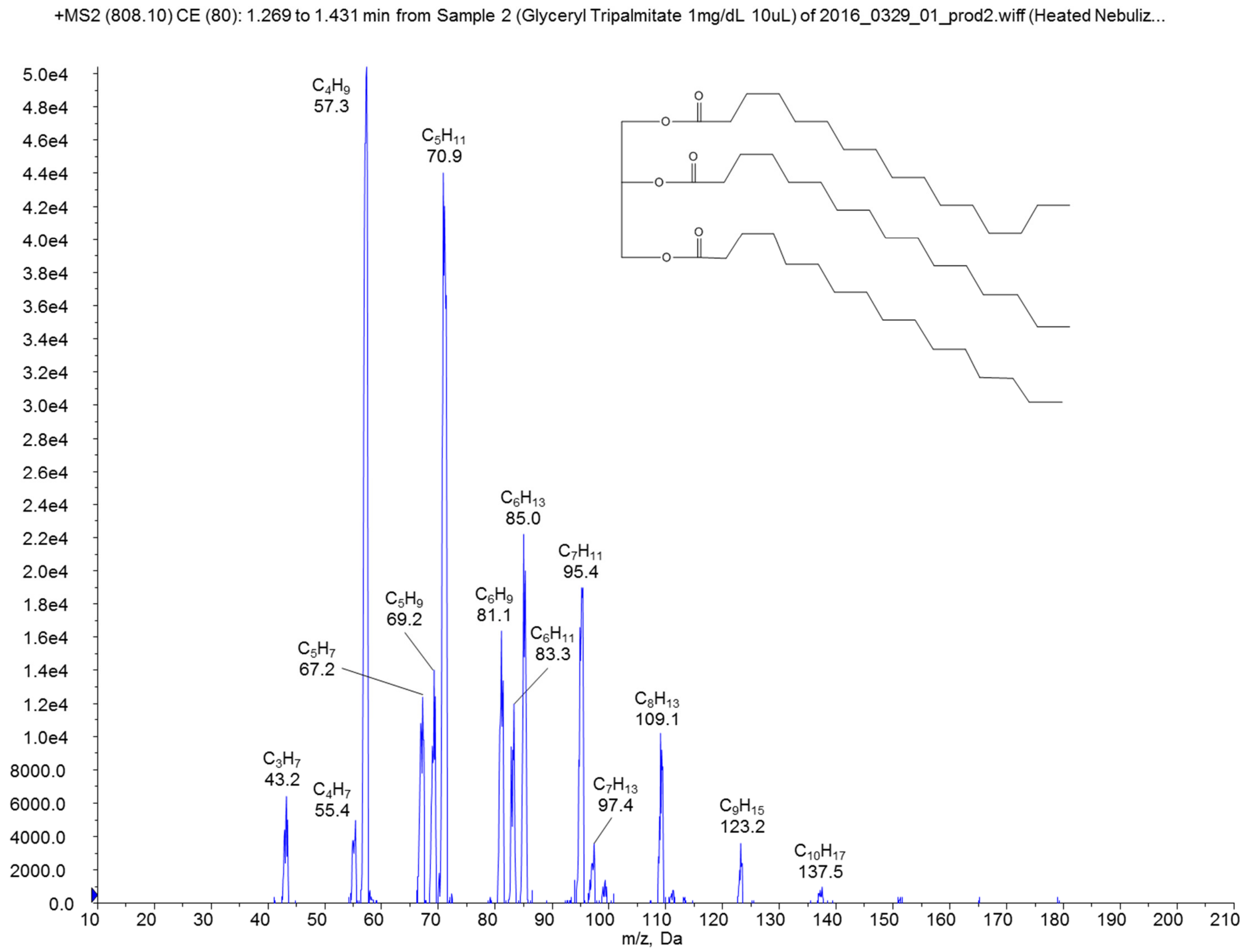

Figure S9. MS/MS of Triolein at CE = 80V. Data summarized in Figure 3 of main text.

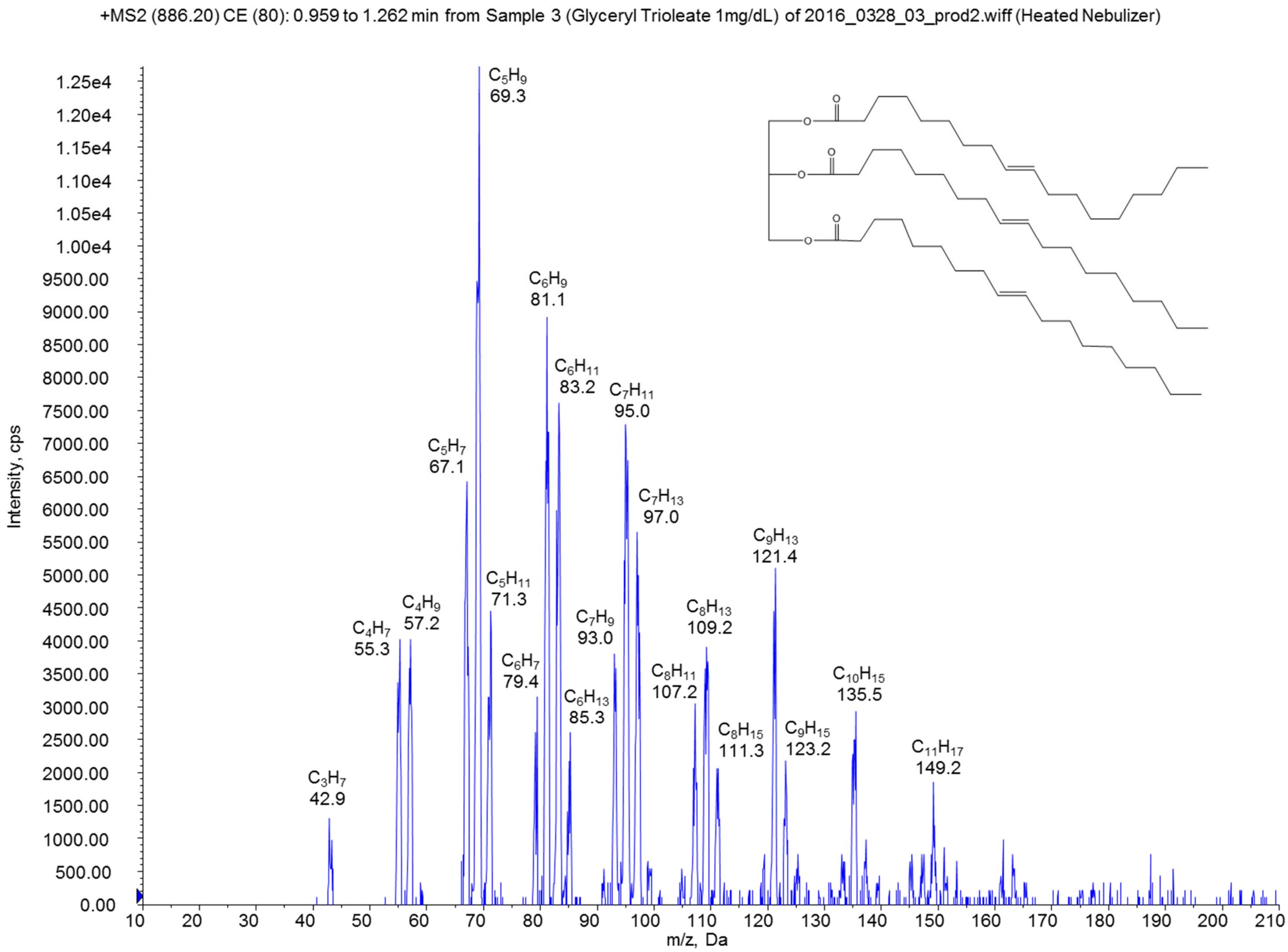

Figure S10. MS/MS of Trilinolein at CE = 80V. Data summarized in Figure 3 of main text.

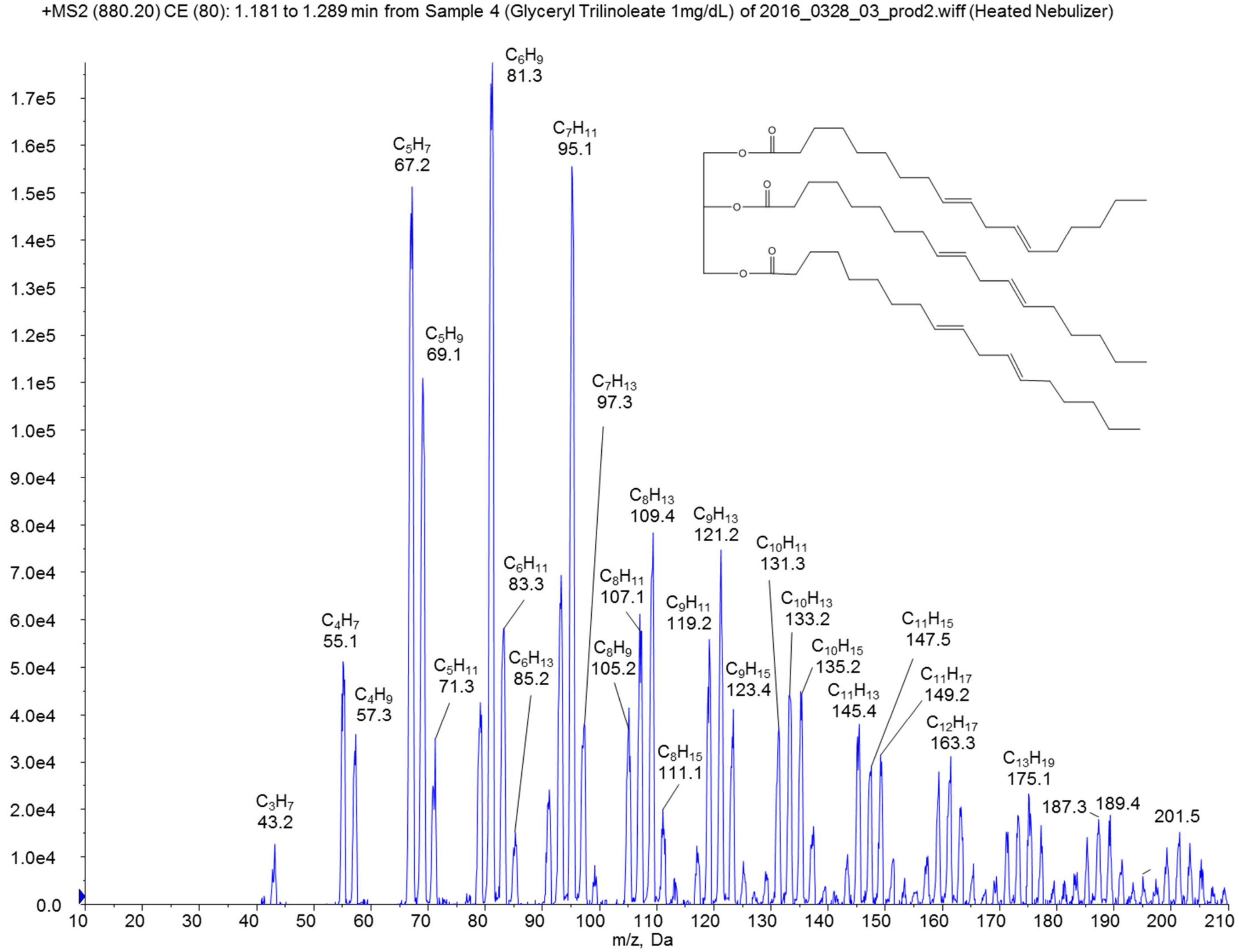

Figure S11. MS/MS of Glyceryl-d5-Triolein at CE = 80V. Data summarized in Figure 3 of main text.

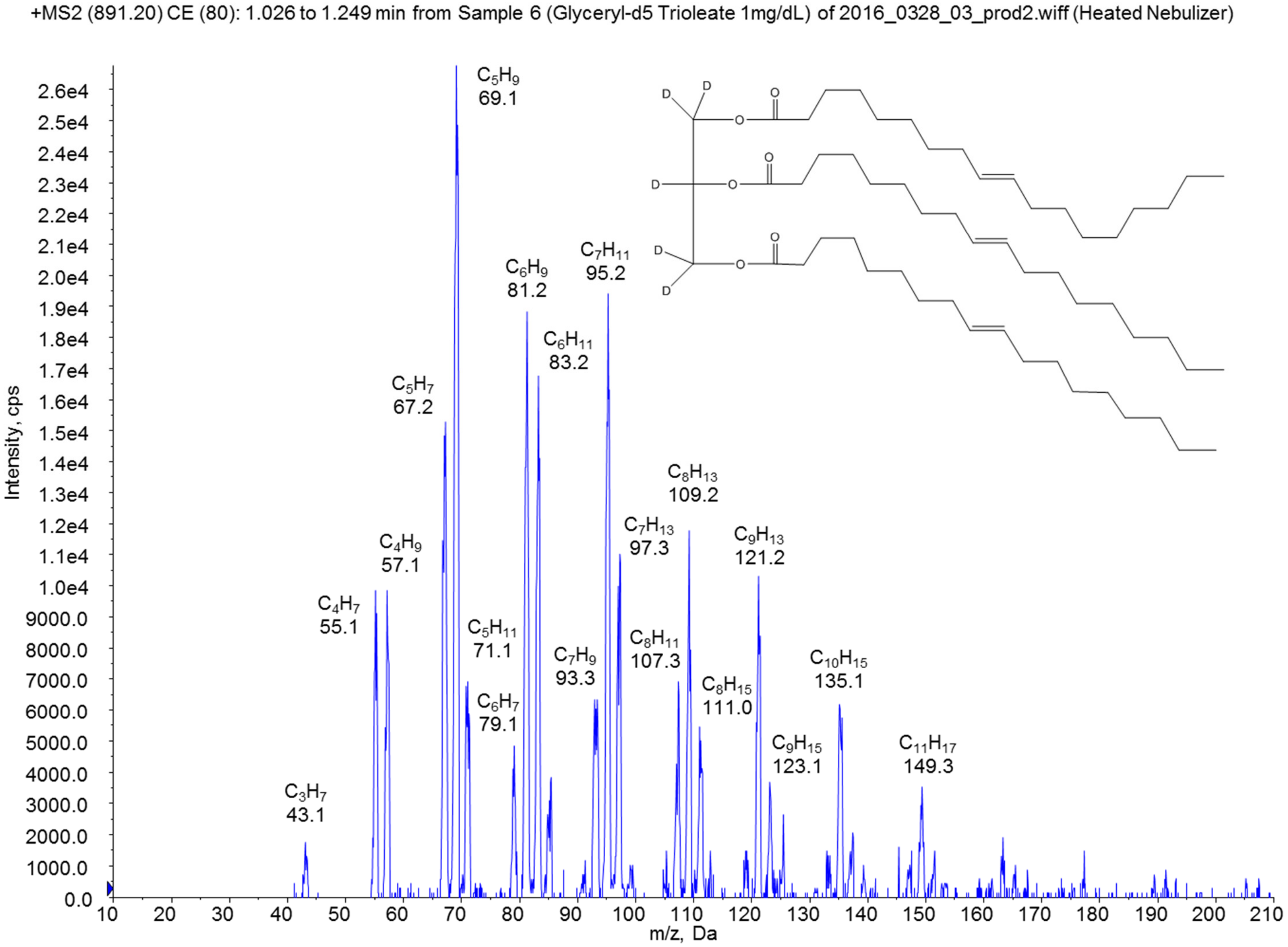

Figure S12. MS/MS of d98-Tripalmitin at CE = 80V. Data summarized in Figure 3 of main text.

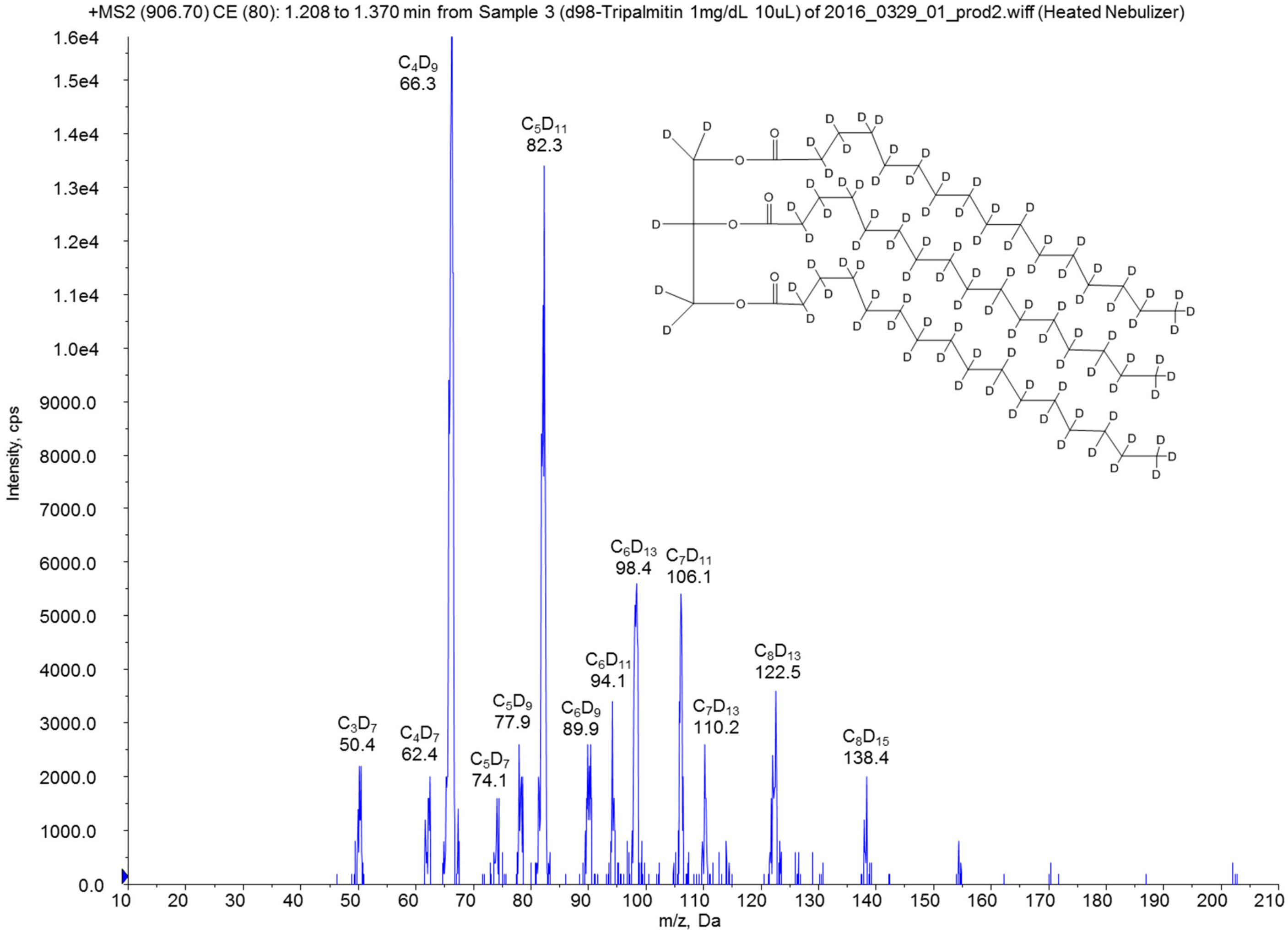

Figure S13. MS/MS of Glyceryl Tri(hexadecanoate-2,2-d2) at CE = 80V. Data summarized in Figure 3 of main text.

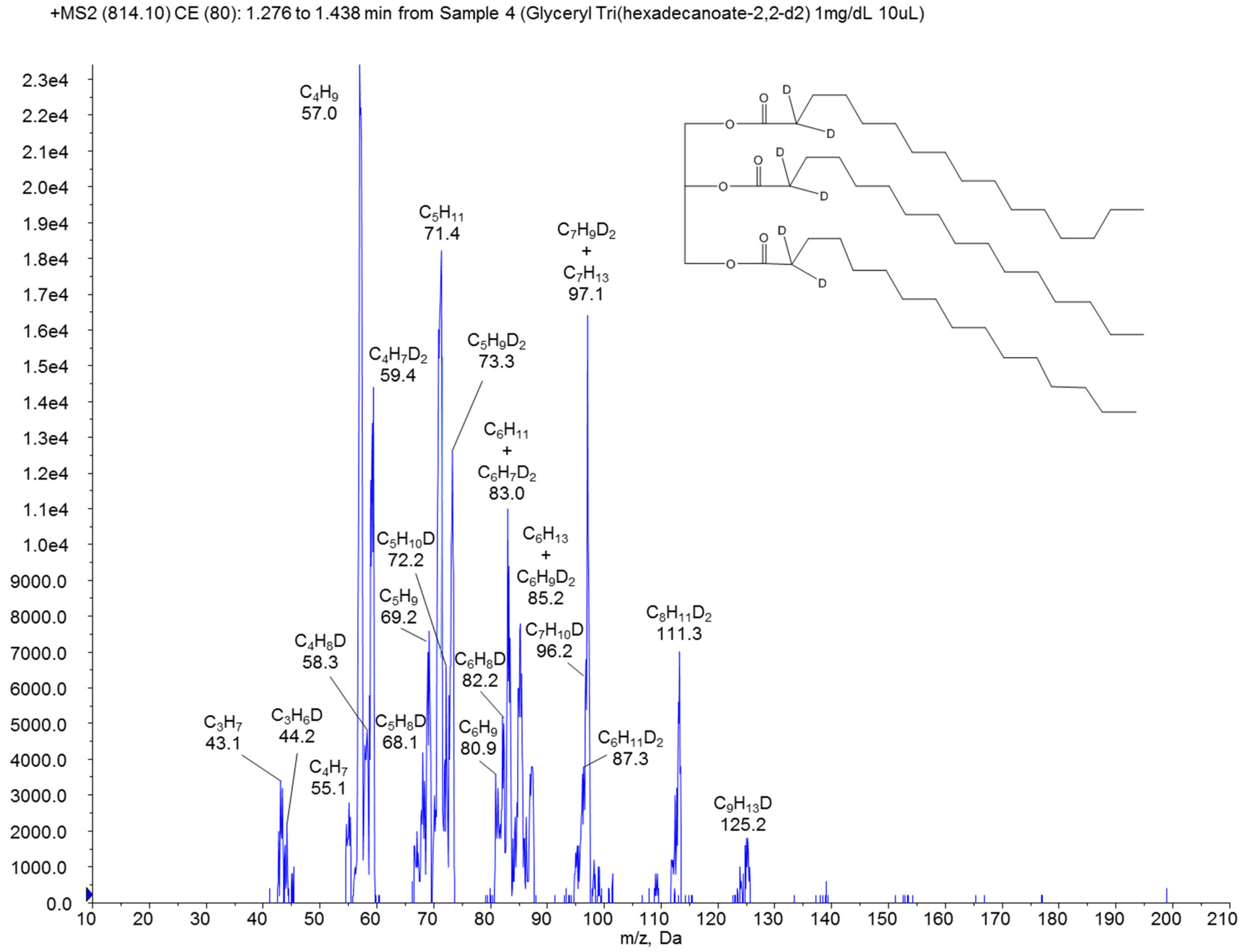

Figure S14. MS/MS of Glyceryl Tri(hexadecanoate-16,16,16-d3) at CE = 80V. Data summarized in Figure 3 of main text.

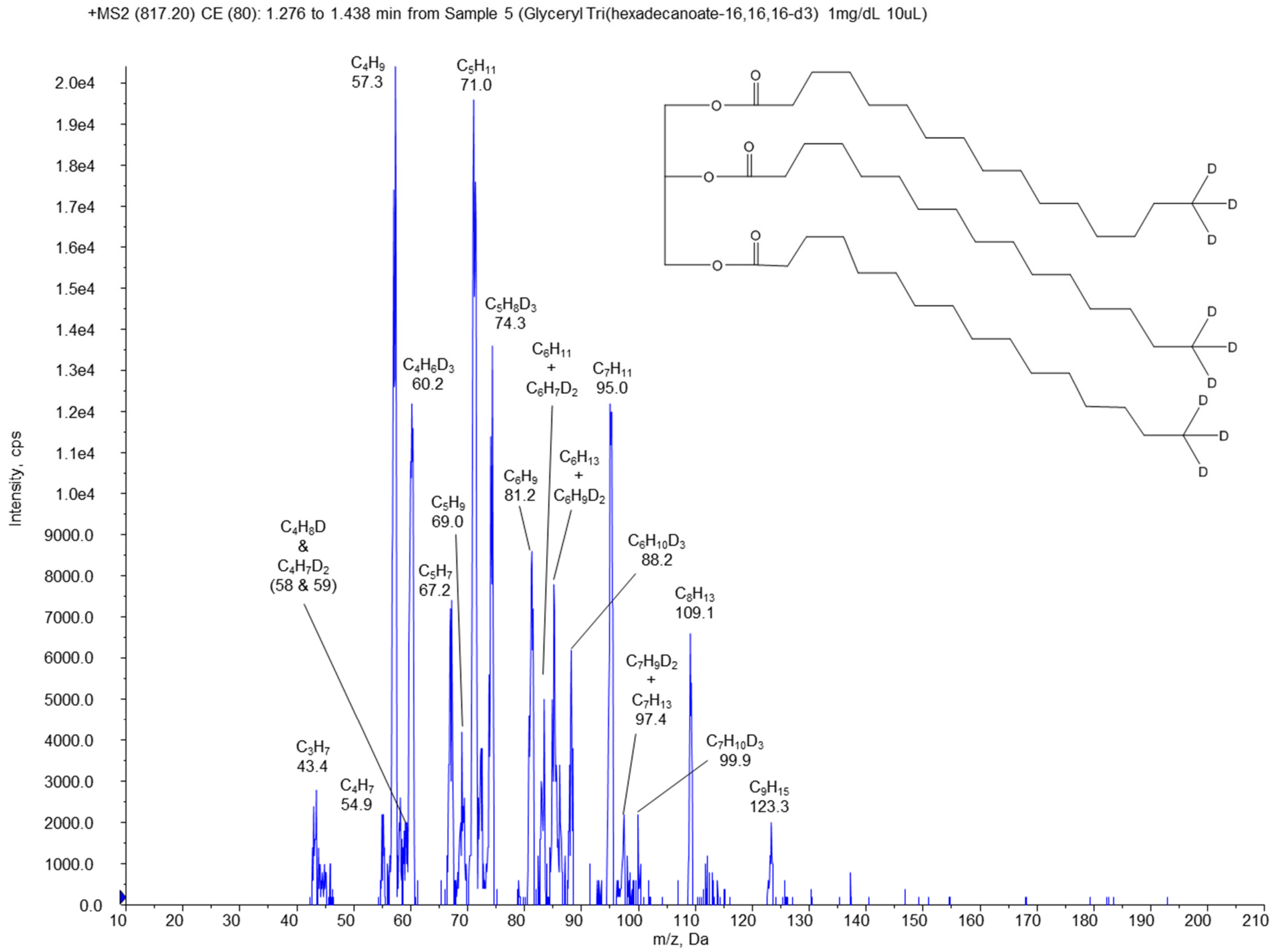

Figure S15. MS/MS of Glyceryl Tri(octadecanoate-18,18,18-d3) at CE = 80V. Data summarized in Figure 3 of main text.

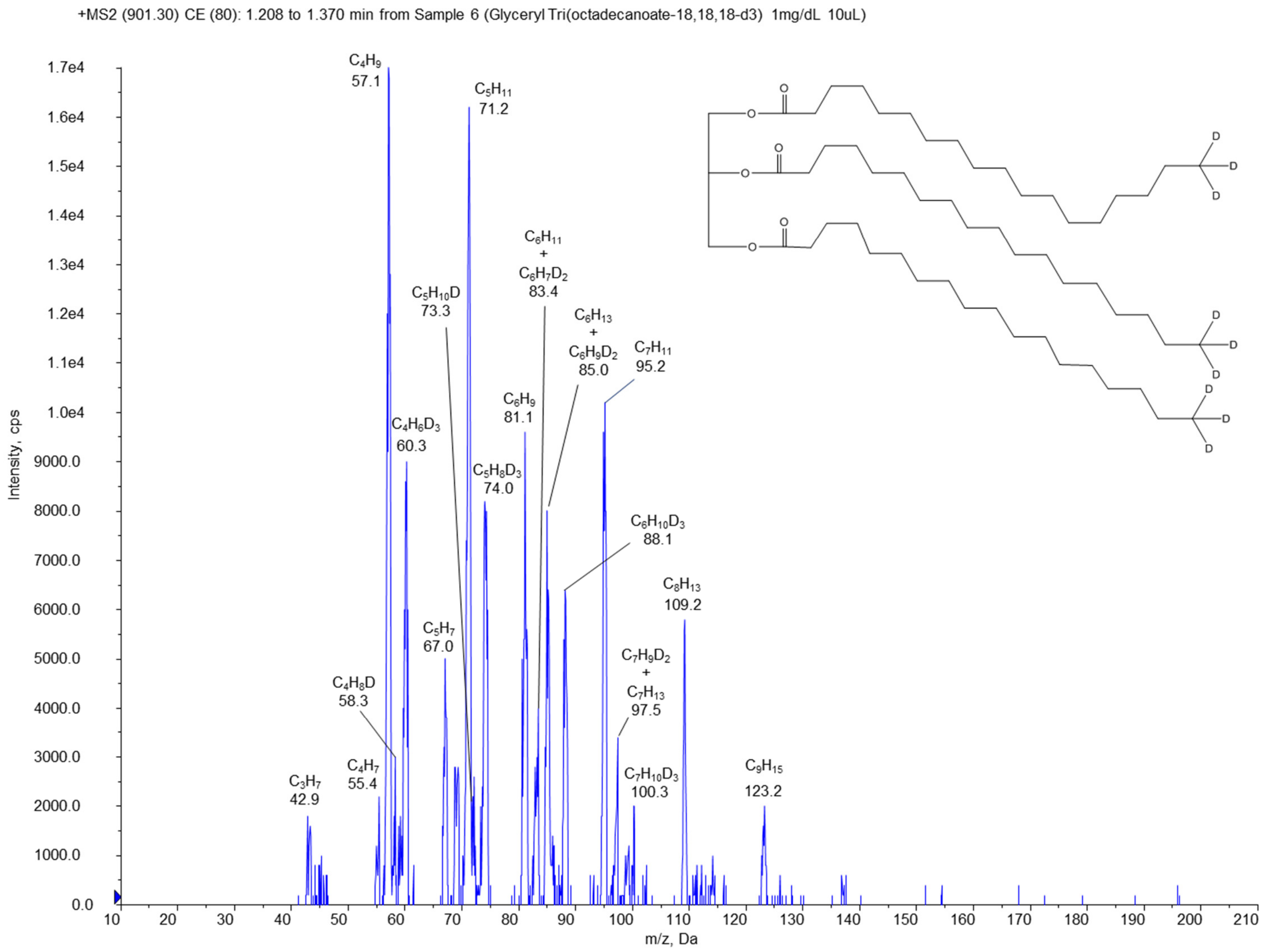

Figure S16. MS/MS of Glyceryl Tri(tetradecanoate-13,13,14,14,14-d3) at CE = 80V. Data summarized in Figure 3 of main text.

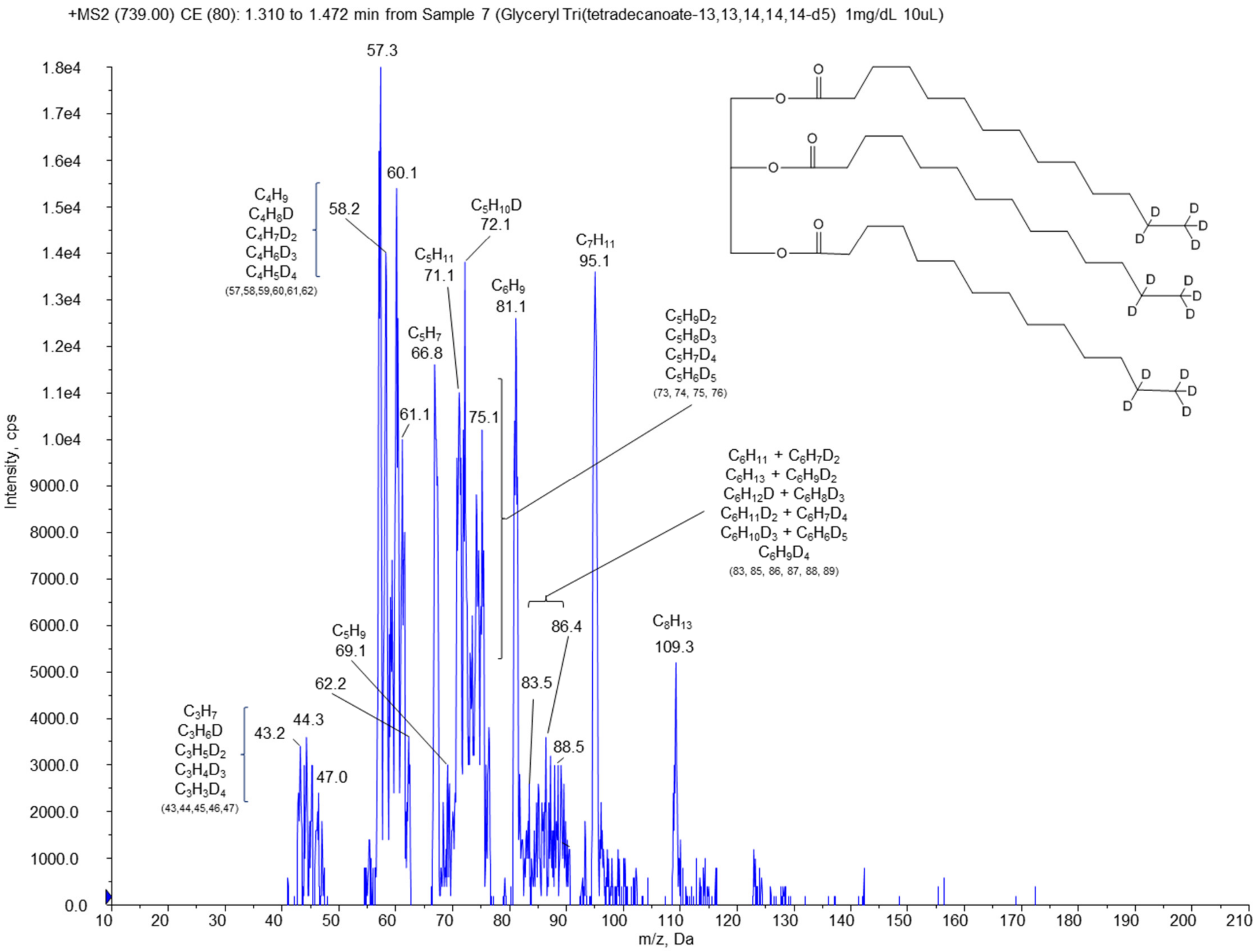

Figure S17. Q1 Scan of Tripalmitin at DP = 262V. Background Subtracted.

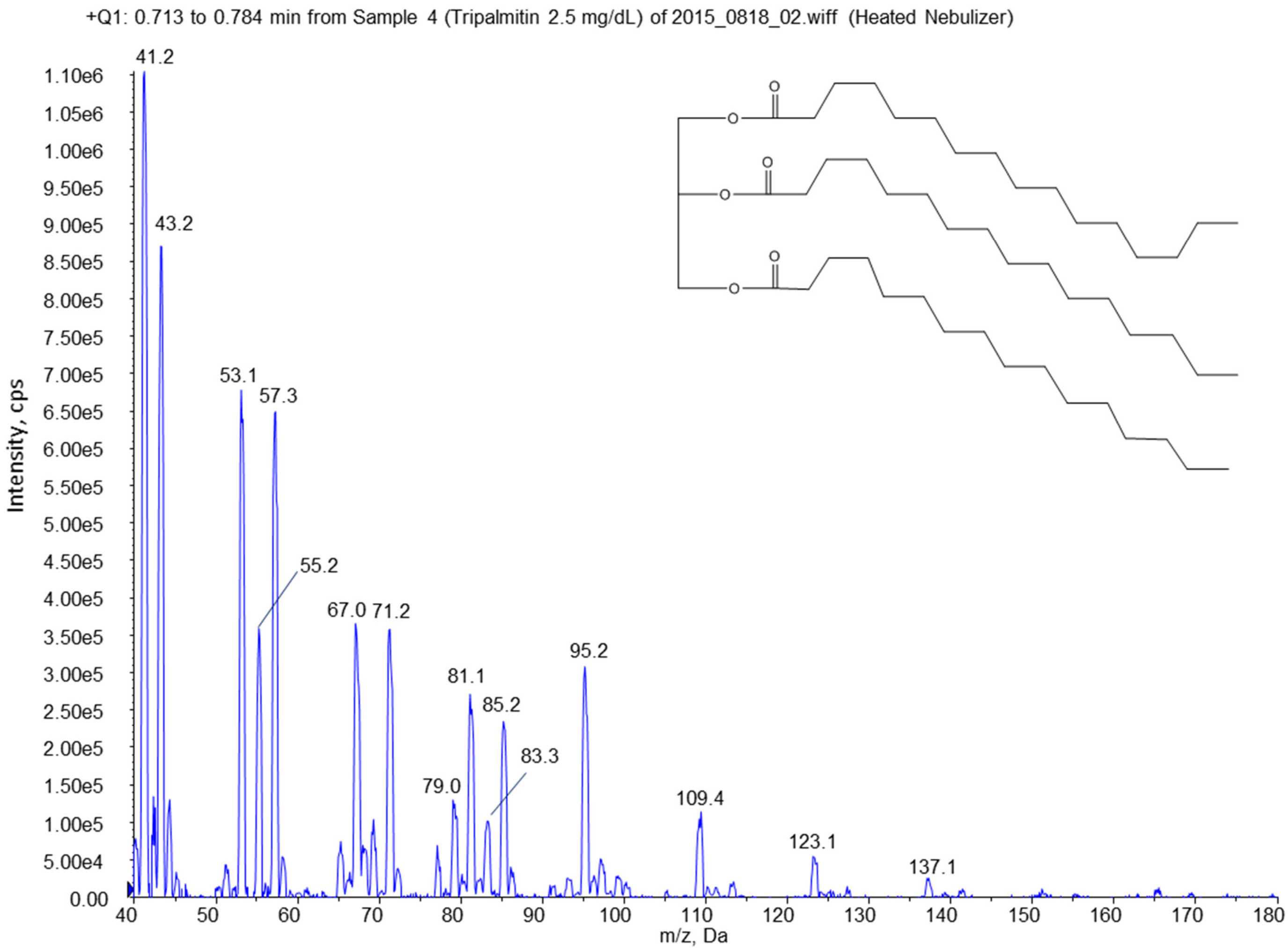

Figure S18. Q1 Scan of Triolein at DP = 262V. Background Subtracted.

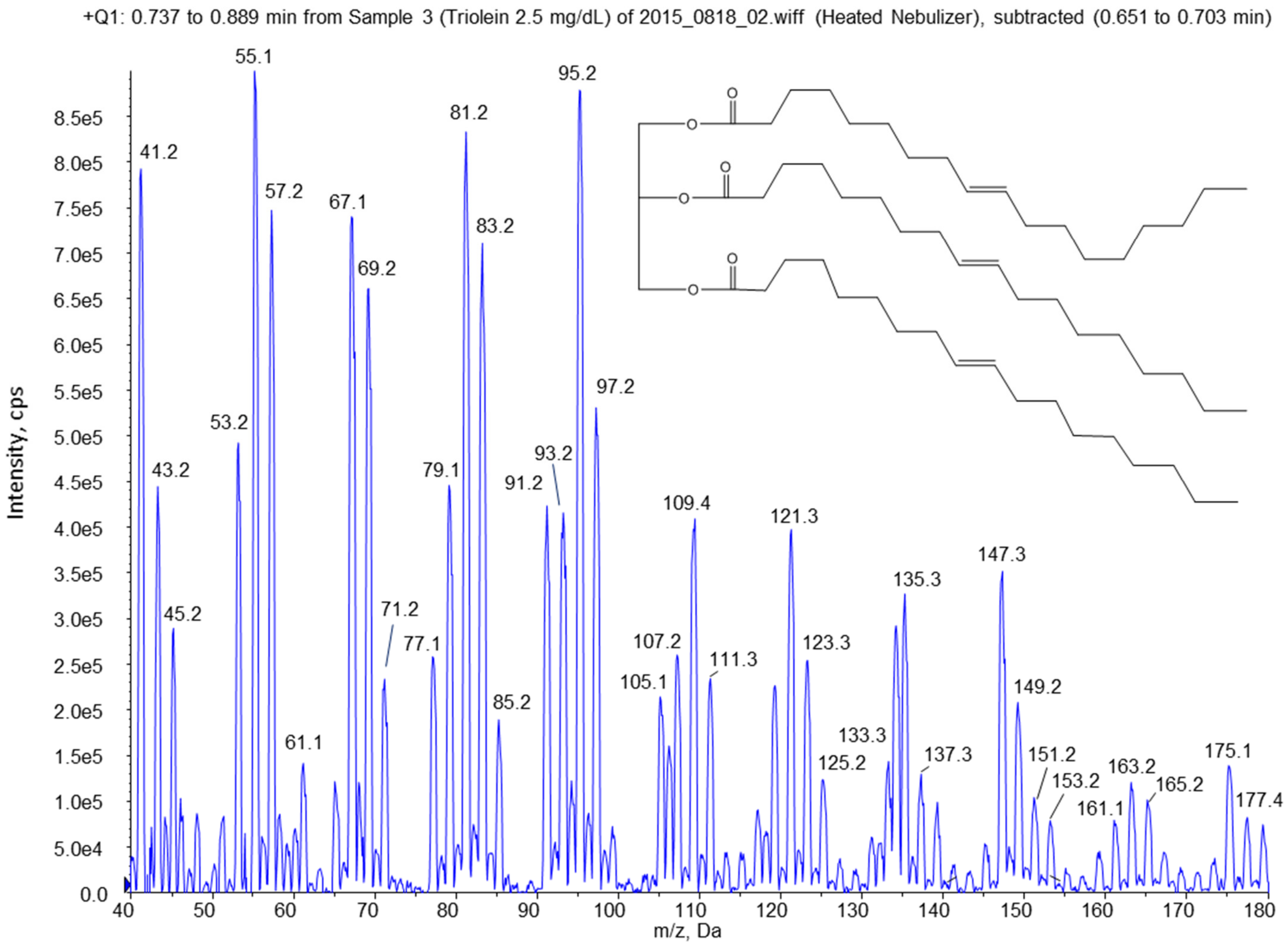

Figure S19. Q1 Scan of Trilinolein at DP = 262V. Background Subtracted.

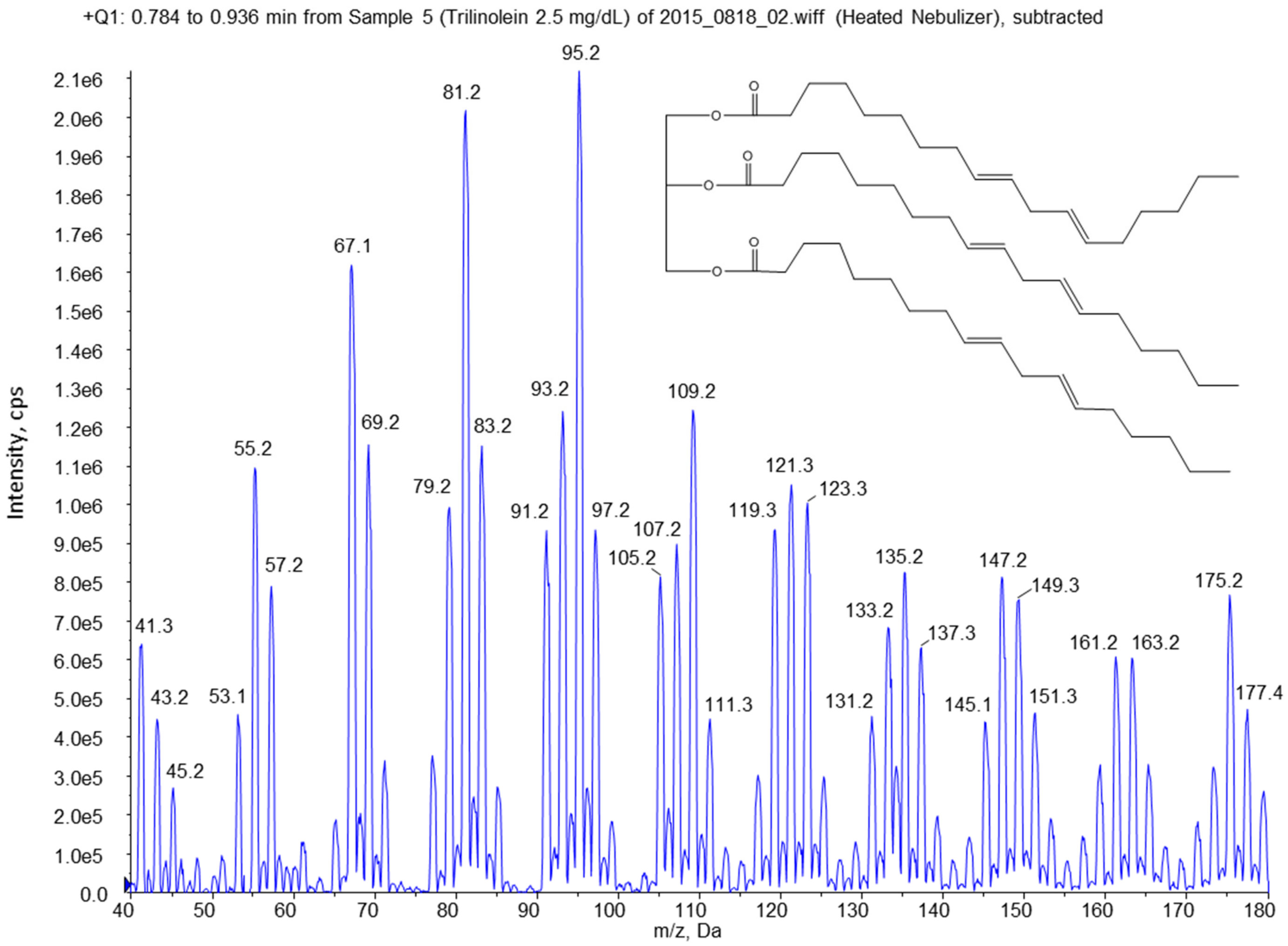

Figure S20. MS/MS of (a) 81 m/z CID fragment from corn oil, averaged over CE of 15V to 78V; (b) 95 m/z CID fragment from corn oil, averaged over CE of 20V to 85V; (c) 98 m/z CID fragment from d98-tripalmitin, averaged over CE of 10V to 55V; (d) 106 m/z CID fragment from d98-tripalmitin, averaged over CE of 15V to 60V.

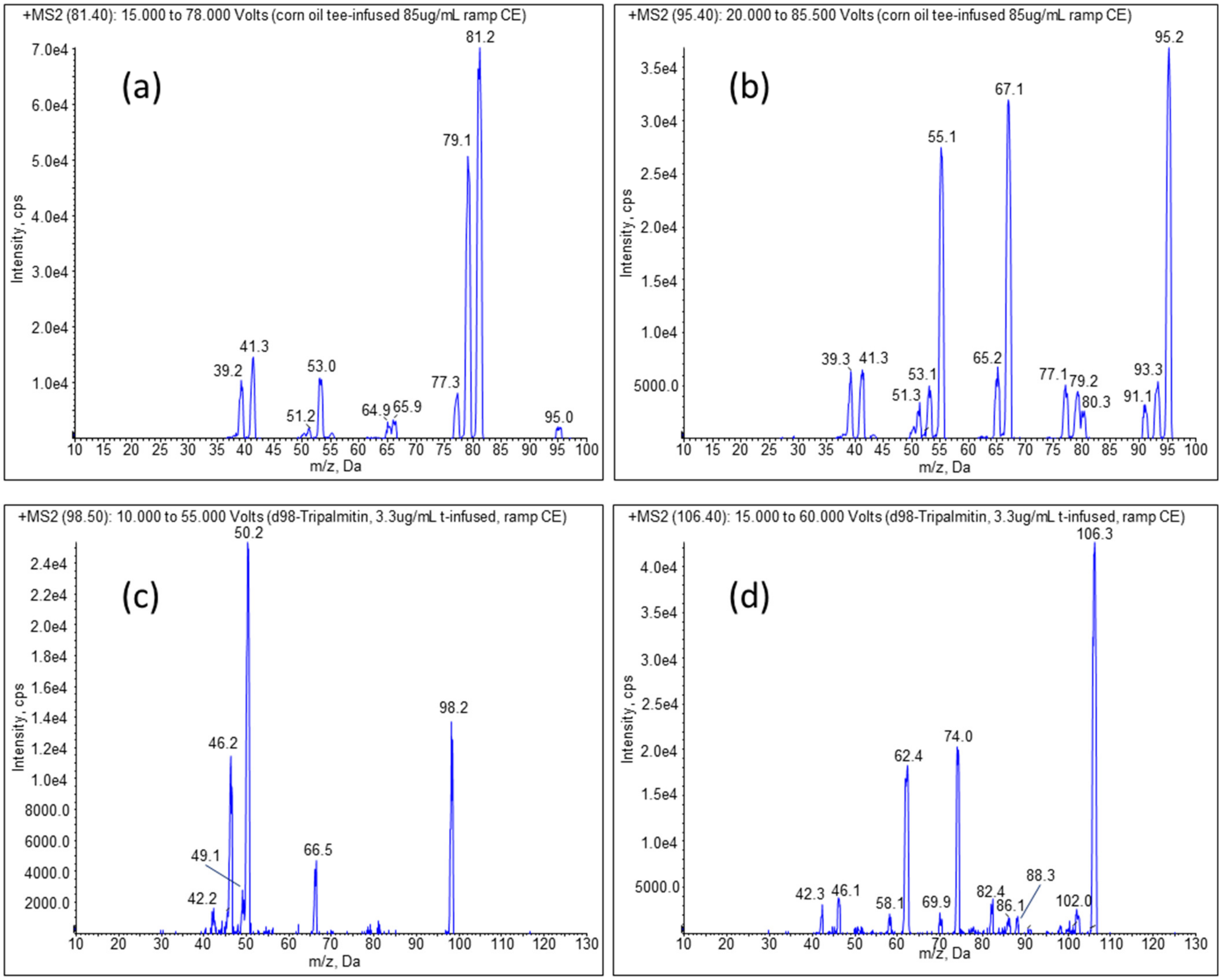

Figure S21: Comparison of response ratios of tripalmitin, triolein, trilinolein showing different efficiency of fragmentation monitoring 95/67 m/z transitions. Also, the response ratio from concentration assigned standard reference materials where the response ratios were normalized for the expected concentration, showing on average the response ratios of the different TG species present in the serum samples were similar. Each error bar is constructed using one standard deviation from the mean.

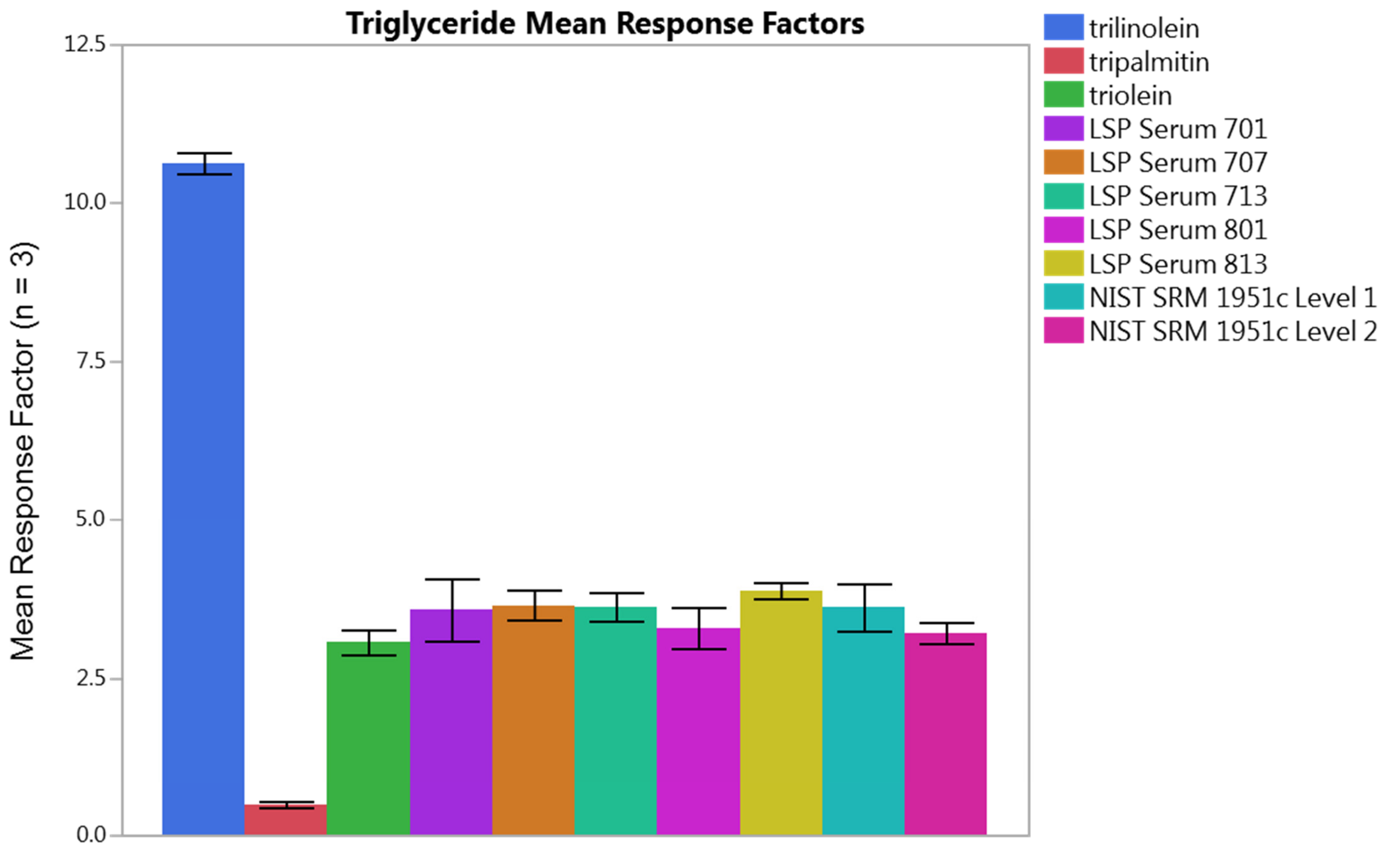

Figure S22. Comparison of different dyslipidemic categories based on lipid ratios. (Error bars represent 95% confidence intervals about the mean.)

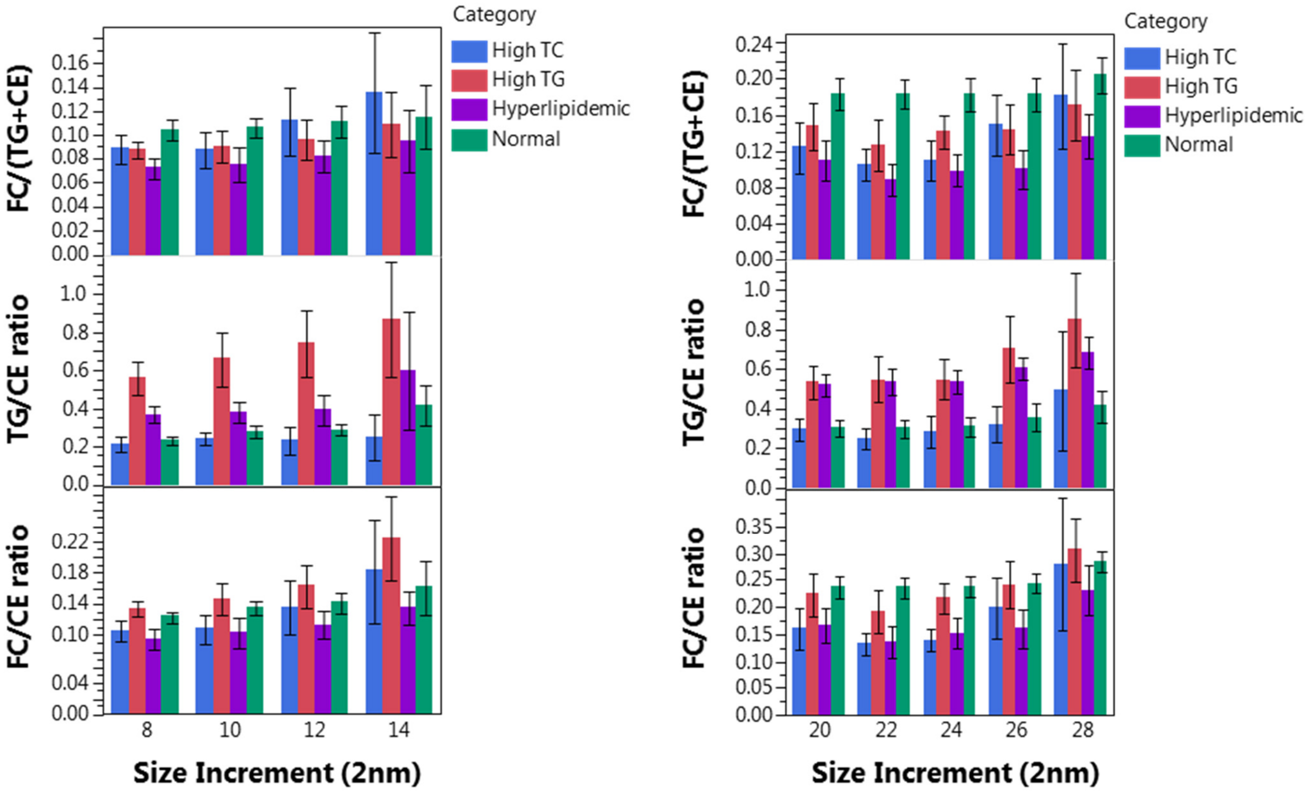

Supplement: Supplementary file 1 — (8.60 MB) [file 13361_2017_1756_MOESM1_ESM.pdf]
